# Functional maturation of human iPSC-derived pyramidal neurons *in vivo* is dependent on proximity with the host tissue

**DOI:** 10.3389/fncel.2023.1259712

**Published:** 2023-11-23

**Authors:** Célia Raïs, Daniela Gaspar Santos, Giulia Sansone, Stéphane Blanchard, Jean-Pierre Bourgeois, Bernd Jagla, Baptiste Saudemont, Laurène Schlick, Stéphanie Pons, Uwe Maskos

**Affiliations:** ^1^Institut Pasteur, Université de Paris Cité, Unité Neurobiologie Intégrative des Systèmes Cholinergiques, CNRS UMR 3571 “Genes, Synapses and Cognition,” Paris, France; ^2^Collège Doctoral, Sorbonne Université, Paris, France; ^3^Institut Pasteur, Université de Paris Cité, Human Genetics and Cognitive Functions, CNRS UMR 3571 “Genes, Synapses and Cognition,” Paris, France; ^4^Institut Pasteur, Université de Paris Cité, Cytometry and Biomarkers Unit of Technology and Service, Center for Translational Science and Bioinformatics and Biostatistics Hub–C3BI, Paris, France

**Keywords:** human induced pluripotent stem cells, neural precursor cells, grafting, neurodevelopment, electron microscopy

## Abstract

Human induced pluripotent stem cells (hiPSCs) have been used extensively *in vitro* to model early events in neurodevelopment. Because of a number of shortcomings, previous work has established a potential to use these cells *in vivo* after transplantation into the mouse brain. Here, we describe a systematic approach for the analysis of transplanted hiPSC-derived neurons and glial cells over time in the mouse brain. Using functional two-photon imaging of GCaMP6f- expressing human neural cells, we define and quantify the embryonic-like features of their spontaneous activity. This is substantiated by detailed electron microscopy (EM) of the graft. We relate this to the synaptic development the neurons undergo up to 7 months *in vivo.* This system can now be used further for the genetic or experimental manipulation of developing hiPSC-derived cells addressing neurodevelopmental diseases like schizophrenia or Autism Spectrum Disorder.

## Introduction

Understanding the development of the human brain is a crucial step toward understanding the function of the adult brain, as well as its disorders. Indeed, many psychiatric diseases diagnosed in childhood, puberty, adolescence, and adulthood, such as Autism Spectrum Disorder (ASD) or schizophrenia (SCZ), have their roots in the development of the brain (Gottesman and Hanson, 2005). While animal models (Molyneaux et al., 2007; Leone et al., 2008; Lui et al., 2011; Gao et al., 2013; Nord et al., 2015) have provided invaluable insights into this process, they do not account for human specificities in brain development. Also, the human “minibrain” organoids have been questioned as experimental models of human brain development (Revah et al., 2022). A review by the Sestan laboratory (Silbereis et al., 2016) highlights the complexity of human development, which reliable *in vivo* models for human-specific neurodevelopmental diseases will have to, at least partially, recapitulate. As compared to non-human primates, the human brain follows a much longer developmental period, extending well beyond adolescence and into adulthood (Gogtay et al., 2004; Tau and Peterson, 2009; Petanjek et al., 2011). Moreover, at the tissue and cellular levels, human cortical development displays added complexity in the proliferative zones and cell types (Bystron et al., 2006; Howard et al., 2008; Fietz et al., 2010; Lui et al., 2011; Dehay et al., 2015). An important step toward recapitulating human brain development has been the introduction of model systems derived from human pluripotent stem cells (hPSCs). Notably, several studies have demonstrated that human embryonic stem cells (hESCs) can mature and differentiate into functional neurons and glia *in vitro* following similar developmental programs as observed *in vivo* (Perrier et al., 2004; Gaspard et al., 2008). Also, the use of human induced pluripotent stem cells (hiPSCs) (Takahashi and Yamanaka, 2006; Park et al., 2008) can circumvent the ethical issues limiting the use of hESCs as they are derived from adult human somatic cells. This technology has already been used *in vitro* to model psychiatric and neurodevelopmental disorders such as ASD and SCZ (Brennand and Gage, 2011). Because of the protracted nature of human development, it remains very difficult to properly address the critical periods for a given disorder such as SCZ or ASD, which are complex, multi-genic disorders of human neural development. It will therefore be critical to match the developmental window under observation to the clinically relevant period for the studied disease. Along that line, several groups have shown that the transplantation of hESCs into the mouse cortex is a useful tool to study mechanisms of human cortical development (Gaillard et al., 1998; Gaspard et al., 2008; Thompson and Björklund, 2015). Espuny-Camacho et al. (2013) transplanted hESC-derived cortical neurons into the newborn mouse brain and showed that they integrated robustly and established defined axonal projections and dendritic patterns corresponding to native cortical neurons. The development, differentiation and maturation of the neurons, as well as the establishment of their connectivity took place over several months (Espuny-Camacho et al., 2013). In a follow-up paper, the same group used the same hESC-derived human Neural Precursor Cells (hNPCs) in an intra-ventricular neo-natal transplantation paradigm in which xeno-transplanted human cortical pyramidal neurons integrated sparsely into the mouse visual cortex and displayed responses to sensory stimuli that resemble those of host neurons, but still followed a protracted development (Linaro et al., 2019). Moreover, the combination of this grafting approach combined with chronic two-photon (2P) imaging provides more information about the development of the human cells inside the mouse brain over extended periods of time, as demonstrated by Real et al. (2018). Using this approach they were able to model early synaptic events in transplanted hiPSCs from two patients with Down syndrome (Real et al., 2018). The chronic observation of the development of such grafted neurons *in vivo* may provide an even more experimentally robust means for discovery. Here, we present a longitudinal study of the development of hNPCs differentiated *in vitro* from hiPSCs, injected into the cortex of newborn mouse pups, and followed for up to 7 months post-injection (mpi). We verified the homogeneity of the population *in vitro* by single cell sequencing, and then injected those NPCs into the brain of newborn mouse pups, to model *in vivo* brain development. We first characterized the development of these cells over time via a histological study and a morphological analysis in 3D. We then used this model to characterize the NPCs’ long-term functional development using chronic calcium imaging *in vivo*, and finally assessed how the interactions between the graft and the host influence this development via histology and electron microscopy (EM).

## Results

**FIGURE 1:**
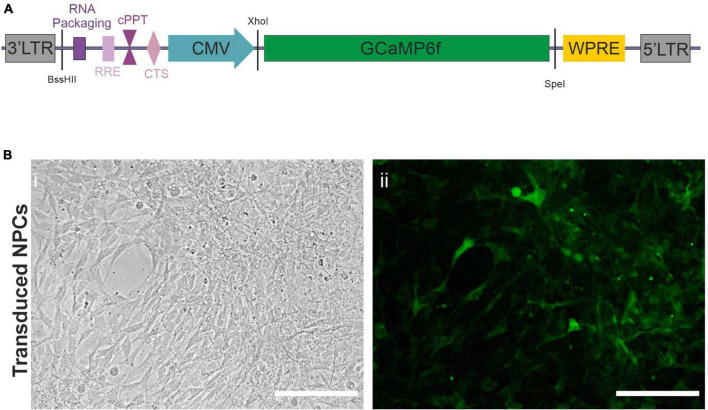
Lentiviral vector. **(A)** Scheme of lentiviral vector construct used to transduce the Neural Precursor Cells (NPCs). LTR, Long Terminal Repeat; RRE, Rev Response Element; cPPT, central Polypurine Tract; CTS, Central Termination Sequence; CMV, Cytomegalovirus Promoter; WPRE, Woodchuck Hepatitis Virus Posttranscriptional Regulatory Element. The restriction sites used to create the virus are also indicated in black. **(B)** Widefield microscope images of NPCs (i) after transduction with the lentiviral vector expressing GCaMP6f (ii). Taken in phase contrast (i), or exhibiting constitutive green fluorescence (ii). Scale bars = 50 μm.

To follow the *in vivo* functional maturation of transplanted hiPSC, we used functional calcium imaging. To this end, we created a lentiviral vector (LV), Figure 1A, as described in section “*Materials and Methods*,” expressing the fluorescent calcium indicator protein GCaMP6f under the control of a CMV promoter. NPCs directed toward an upper-layer cortical fate were obtained from a previously characterized hiPSC line. This developmental stage requires a minimal amount of transcription factors while engaging the cells in the forebrain lineage, and has been shown to develop into cortical pyramidal neurons by default *in vitro* (Gaspard et al., 2008) and *in vivo* (Espuny-Camacho et al., 2013) giving us access to early steps of cortical development in the context of a living brain with minimal experimenter intervention. We were using a differentiation protocol as described in *Materials and Methods*. Briefly, hiPSC were induced into the neuroectodermal lineage via dual smad inhibition and kept in a neuronal medium in the presence of proliferation factors to maintain the NPC state. Early-passage NPCs were then transduced with the LV as described in *Materials and Methods*. Figure 1B shows an example of a transduced culture.

### hiPSC are differentiated into a homogeneous population of NPCs *in vitro*

**FIGURE 2:**
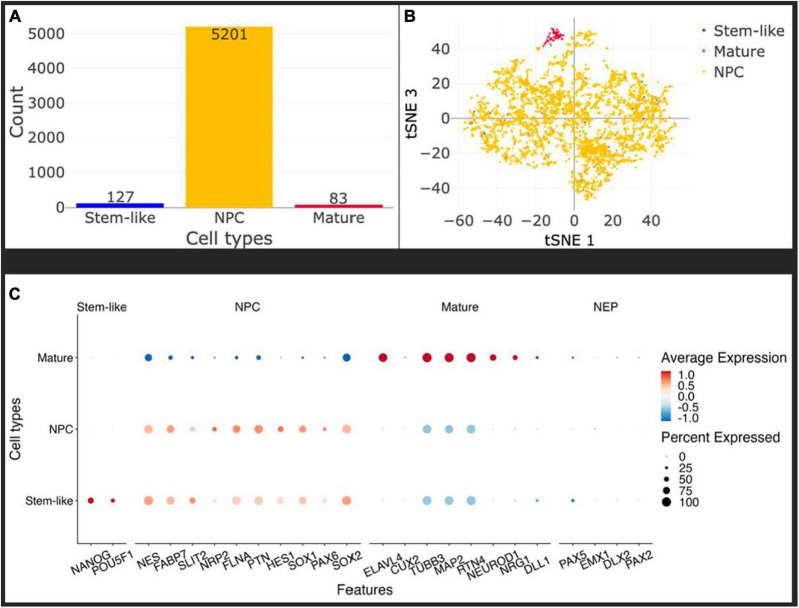
*In vitro*, the injected cells represent a homogeneous population of Neural Precursor Cells (NPCs). **(A)** Histogram of identified cell types: stem-like (blue), mature (red), NPC (yellow). Only about 1.5% of cells have some mature characteristics and a maximum of 2.3% can be labeled stem-like cells, leaving more than 96% of the cells as NPCs. Cell types were identified using the Seurat cluster algorithm implemented in SCHNAPPs and the expression of marker genes within them **(C)**. Stem-like cells are cells expressing at least one UMI of either NANOG or POU5F1. **(B)** Projection of the cells on the first and third t-SNE dimensions shows that the stem-like cells colocalize with the NPC whereas the mature cells form a distinct cluster. **(C)** Dot plot of cell-type specific marker genes. Standardized expressions are color coded, showing high expressions in red and low expression in blue. The size of dots corresponds to the percentage of cells expressing that gene per group of cells. Stem-like cells are defined by the presence of at least one UMI of NANOG or POU5F1. Those cells mostly express NPC marker genes at a similar level as the bona-fide NPCs indicating they might as well be NPCs.

To control for the homogeneity of the NPC population after differentiation *in vitro*, and account for the developmental stage of the cells before injection, we performed single-cell sequencing on NPCs at passage 11 using 10 × Genomics technology as described in *Materials and Methods*. We did not remove any cell from the analysis based on usual quality criteria like mRNA content or number of UMIs to ensure to capture all cell populations possible, see Figure 2A. Using SCHNAPPs (Jagla et al., 2021), we performed normalization, dimension reduction, and cluster analysis that gave closely related groups of cells that were identified as NPCs of which one somewhat dissociated group resembled mature neurons. The clusters were combined and annotated based on the expression profiles discussed below into “stem-like,” NPC, and mature. We used minimal thresholds for identifying possible stem-like cells by expression of NANOG or POU5F1 (Na et al., 2010). These cells are otherwise evenly distributed in the tSNE space (Figure 2B) and do not form a defined group of cells indicating that these cells might belong the NPC group, too. Importantly, all clusters expressed Nestin (NES), an established marker of NPCs, as well as FABP7 (Matsumata et al., 2016), which collectively point toward a *bona fide* NPC phenotype (Figure 2C; Boissart et al., 2013; Espuny-Camacho et al., 2013). The neuro-epithelial (NEP) markers Pax5 and Sox1 were expressed in all clusters at low expression levels (Figure 2C), but not the other selected NEP markers EMX1, DLX2, and PAX2. Moreover, the markers related to a mature neuronal phenotype like ELAVL4 and Cux2 were not expressed, indicating that our injected population of cells is a homogeneous population of NPCs. Of note is the expression of TUBB3 and MAP2, which are commonly expressed in mature neurons, but are also expressed to a lesser extent in neuronal progenitors in the mouse (Charrière-Bertrand et al., 1991; Menezes and Luskin, 1994). An exception is the cluster assigned mature, which is particularly enriched in TUBB3-expressing cells, as well as ELAVL4, suggesting a more developed phenotype. Overall, these data demonstrate that the injected cells are a homogenous population of NPCs, with a small number of cells slightly more engaged in the neuronal differentiation process.

### Grafted NPCs integrate and mature in the upper layers of mouse cortex

A suspension of 200,000 cells per μl was injected into the neonatal mouse brain as described previously (D’Alessio et al., 2020). To assess the quality of the injection, and the proper grafting of the cells, injected mouse brains were sampled 1-, 2-, 3-, 4-, 5-, and 6-months post-injection (mpi). After perfusion, brain sections were prepared as described in *Materials and Methods*. Since the human cells express GCaMP6f, they were stained with an anti-GFP antibody for further analysis.

**FIGURE 3:**
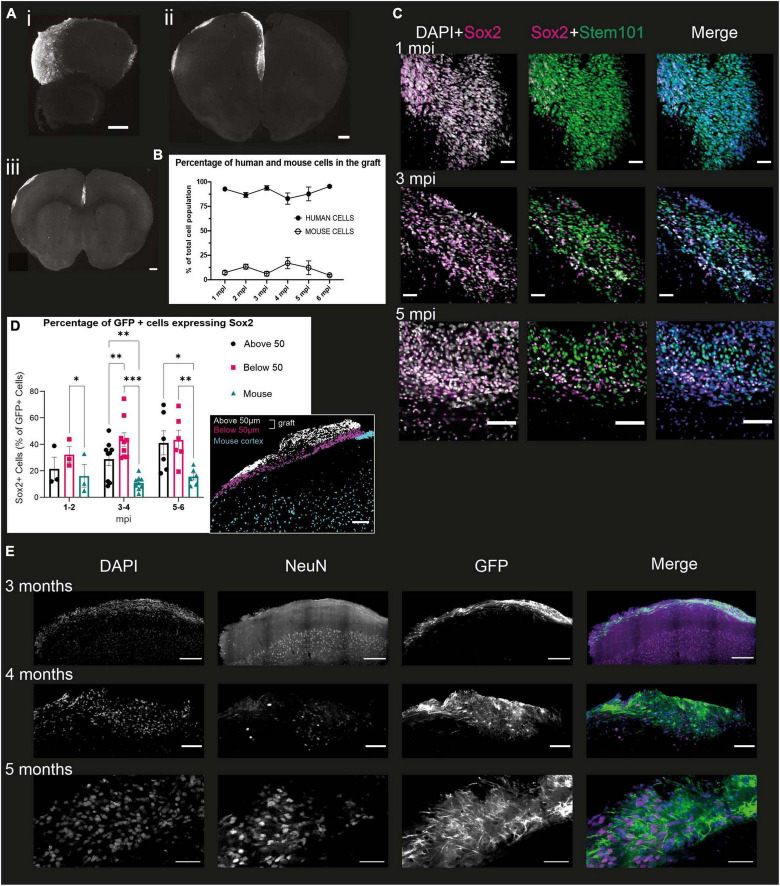
Grafted NPCs exhibit protracted differentiation into post-mitotic neurons. **(A)** Immunofluorescence staining of mouse brains at 6 mpi, with representative slices of the most rostral (i), the central (ii) and most caudal (iii) slices. Scale bar = 500 μm. **(B)** Percentage of the grafted cell population (based on DAPI staining) identified as human (STEM101-positive) or mouse (STEM101-negative) in the Icy Software (*n* = 34 mice). **(C)** Immunofluorescence staining of mouse brains 1, 3, and 5 mpi, with antibodies directed against Sox2, a multipotency marker, STEM101, a human nucleus-specific antibody, and DAPI. The DAPI + Sox2 panel shows the total number of Sox2 cells in the graft, while the Sox2 + STEM101 panel shows human cells that are positive for Sox2. Scale bar = 50 μm for 1 and 3 mpi and 100 μm for 5 mpi (*n* = 18 mice). **(D)** Quantification of the proportion of human Sox2 + cells at different time points of development in the mouse cortex and “above” or “below” the 50 μm limit from the graft-mouse border. Represented is the mean for all brains of a given time point and the SEM. One symbol represents one mouse. Two-way ANOVA and Dunn multiple comparison, *p < 0.05, **p < 0.005, ***p < 0.001 (*n* = 18 mice). Insert: representative grafted brain with subdivisions as obtained from the analysis. Scale bar: 100 μm. **(E)** Immunofluorescence staining of mouse brains 3, 4, and 5 mpi with antibodies directed against NeuN, a marker for post-mitotic neurons, GFP, a proxy for the detection of the human cells, and DAPI. Scale bar = 200 μm for 3 mpi, 100 μm for 4 mpi, and 50 μm for 5 mpi (*n* = 11 mice).

As early as 1 mpi, the transplanted cells integrate into the upper parts of the mouse brain (Figures 3A i–iii). The graft extends over several hundred microns along the rostro-caudal axis and covers all of the cortex along the medio-lateral axis (Figures 3A i–iii). The proportion of brains where grafted cells were visible in the cortex for each time point is 51.3% (± 15.2%). The remaining brains either had no visible graft, or had a visible graft in the ventricle, suggesting that the injection was too deep to target the cortex. The human cells are densely packed and do not appear to have a layered organization.

We then evaluated the differentiation of the human cells within the cortical graft over 6 mpi. First, we assessed the proportion of cells within the graft that were indeed human cells, and whether the proportion of human cells changed throughout time. For this purpose, we quantified the amount of DAPI positive cells which were also stained with STEM101, a human-specific antibody directed against Ku80, within the defined “graft” ROI (region of interest). This ROI is manually defined as the area containing all of the STEM101 nuclei above layer 1 of the mouse cortex (detailed in *Materials and Methods*). We found that the proportion of human cells in grafts is 89.2% ± 5.7% at all time-points and does not significantly differ over time (Figure 3B), indicating that only a small proportion of mouse cells do take part in the graft, which is therefore a model that lets us mainly observe the development of human cells in the context of a living mouse brain.

To understand further the level of differentiation of the human cells in the first few months, we co-stained 50 μm slices from grafted brains with STEM101 and Sox2, a marker of multipotency notably expressed in radial glial cells (Ellis et al., 2004; Figure 3C). From this staining, we could observe four different populations of cells: human cells, which can be either Sox2-positive (Sox2 +) or Sox2-negative (Sox2-), and mouse cells, which can also be Sox2 + and Sox2-. We observed that at all time points, the proportion of human cells expressing Sox2 (66.6% ± 10.3%) is significantly higher than the proportion of mouse cells expressing Sox2 (31.7% ± 10.7%), demonstrating that the human cells are at an earlier stage of differentiation compared to the mouse cells.

To verify if the proximity of the more differentiated mouse cells could impact the development of the human cells, we separated the human cells into three compartments for further quantification. The first compartment corresponds to the mouse cortex, below the limit of the human somas, the second compartment was considered from this limit to 50 μm above it (“below 50 μm”), and the last from the 50 μm limit up to the surface of the cortex (“above 50 μm”) (Figure 3D insert). We selected a 50 μm distance from the mouse cortex as we estimated that this was likely to be a long enough distance to limit direct cell-cell interactions, while letting us include smaller grafts in the study. While there is no significant difference in the number of Sox2 + GFP-expressing human cells in each compartment before 2 mpi, at the 3 to 4 months timepoint we see that the mouse compartment has a significantly lower proportion of Sox2-expressing human cells compared to the “Below 50 μm.” This difference is not significant between the mouse compartment and the “Above 50 μm,” which displays a very large variability. However, at the 5 to 6 mpi timepoint, the proportion of Sox2-expressing GFP + cells in the “Above 50 μm” becomes significantly larger than in the “Below 50 μm” compartment. These results corroborate the hypothesis that the rate of development of the grafted human cells is influenced by their distance to the part of the mouse cortex that was able to develop in an expected manner, i.e., at a much faster pace than the human cells, delimited by a very distinct first cortical layer, observable with the DAPI staining (Figure 3D).

To analyze whether the grafted human cells did indeed differentiate into neurons, we stained 50 μm slices of grafted brains with antibodies directed against GFP to identify the human cells, and NeuN, a post-mitotic neuron marker (Mullen et al., 1992). This was performed on brains sampled every month from 1 to 6 mpi. On all the brains stained, we could observe NeuN positive nuclei within the graft from 4 mpi, and were only able to observe NeuN-positive nuclei with a GFP-positive soma from 5 mpi. In contrast, the mouse cortex exhibited a strong NeuN staining as early as 1 mpi (Figure 3E), consistent with the mouse cortex having reached maturity. This suggests that the grafted NPCs do differentiate into neurons, but at a much slower pace than the mouse cells, in line with what has been shown previously (Espuny-Camacho et al., 2013; Linaro et al., 2019; D’Alessio et al., 2020).

**FIGURE 4:**
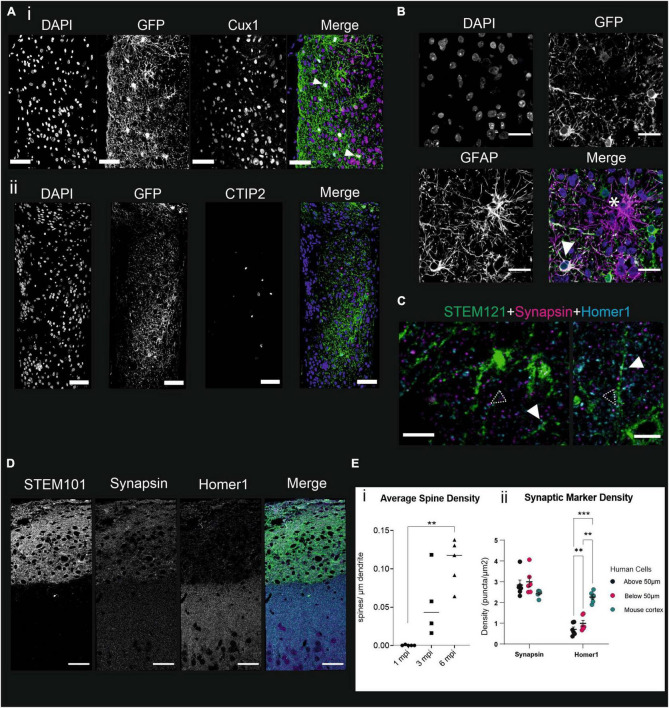
Grafted NPCs differentiate into layer 2/3 cortical neurons with immature synapses, and into astrocytes at 6 mpi. **(A)** Immunofluorescence staining of mouse brains 6 mpi, nuclei marked with DAPI, and antibodies directed against GFP to identify the human cells. (i) Cux1, a marker expressed in layer 2/3, white arrows represent Cux1 + cells with a visible GFP positive soma, or (ii) CTIP2, a marker expressed in layers 4–6. Scale bar = 50 μm. **(B)** Immunofluorescence staining of mouse brains 6 mpi with antibodies directed against GFP and GFAP to mark astrocytes. The star shows a cell expressing GFAP but not GFP, suggesting it is a mouse astrocyte while the white arrowhead points to a GFP + astrocyte. Scale bar = 20 μm. **(C,D)** Immunostaining of mouse brains 7 mpi with cell nuclei marked with DAPI and antibodies directed against Homer1, a post-synaptic marker, synapsin, a pre-synaptic marker, and STEM121, specific to human cytoplasm. Filled arrowheads point to Homer1 expression at putative human dendritic spines, dashed arrowheads point to synapsin-Homer1 juxtapositions. **(C)** Scale bar = 5 μm, **(D)** Scale bar = 50 μm. **(E)** (i) Quantification of the average spine density per μm of dendrite at 1, 3, and 6 mpi. Kruskal–Wallis multiple comparison test, **p < 0.01, ***p < 0.001. (ii) Quantification of the density of puncta per μm^2^ for Synapsin and Homer1 stainings in defined compartments of the mouse cortex. Wilcoxon test, *p < 0.05, ns = not significant.

To examine further the differentiation of the grafted NPCs, we performed immunostainings of 50 μm slices of grafted brains at 6 mpi, with antibodies directed against the cortical layer 2/3 marker Cux1 and layer 4/6 marker CTiP2 (Molyneaux et al., 2007). Cux1 is expressed by most cells in the graft, the arrow points to a GFP-positive cytoplasm with a Cux1 + nucleus (Figure 4A i). On the other hand, the CTiP2 staining is sparser, and we were not able to find GFP + cells with a CTiP2 + nucleus (Figure 4A ii), indicating very few if any human cells express this protein at 6 mpi. This shows that the grafted NPCs differentiate mainly into upper layer cortical neurons at 6 mpi.

Neural Precursor Cells have been shown *in vitro* and *in vivo* to give rise to both neurons and astrocytes (Culican et al., 1990; Anthony et al., 2004; van den Ameele et al., 2014; Gouder et al., 2019). To assess whether the grafted NPCs differentiate into astrocytes as well, we stained grafted brains 6 mpi with antibodies directed against GFAP, an astrocyte marker, GFP, to identify the human cells, and DAPI to mark the DNA. The first striking feature that we could observe was that the density of astrocytes is much higher in the graft than in any other area of the mouse cortex (Supplementary Figure 1). Within the graft, both human and mouse astrocytes can be observed, which shows that the grafted NPCs do differentiate in part into astrocytes (Figure 4B). These observations lead us to conclude that the grafted NPCs differentiate into upper layer cortical neurons and astrocytes, in a protracted manner.

### Grafted NPCs form spines and synapses 7 months after injection

To assess the presence of synapses in the graft, we stained brains that had undergone 2P imaging, with antibodies directed against the pre-synaptic marker Synapsin (Gitler et al., 2004), and the post-synaptic marker Homer 1 (Ango et al., 2000; Xiao et al., 2000; Figures 4C, D), at 7 mpi. Both markers were present in the graft at that time point. Human dendrites could be observed with Homer/Synapsin complexes (dashed arrowheads) both on the dendritic shaft and on dendritic spines (Figure 4C). Homer 1 expression can also be observed on its own on human dendritic spines (Figure 4C, filled arrowheads).

To assess the dendritic maturation of the NPCs into neurons, we performed a morphological analysis using the DIVA software, a virtual reality software (El-Beheiry et al., 2020). Isolated pyramidal neurons were selected based on morphology and imaged with a confocal microscope. The dendrites were manually traced using the “ruler” tool, and dendritic spines were quantified using the “counter” tool from DIVA. Only primary dendrites were considered, and on these dendrites all spines were taken into account. We did not discriminate between spines based on their morphology. We measured a significant increase in the density of spines/μm of dendrites between 1 mpi (0.00028 ± 0.00055) and 6 mpi (0.11 ± 0.027) (Figure 4E i) as well as changes in the average distance from the soma, which were all not significant.

We therefore wanted to measure the maturity of synapses formed by the human cells at 7 mpi. We quantified the density of signal for Homer 1 and Synapsin markers using the Icy software (De Chaumont et al., 2012) spot detector, in the mouse, “Above 50 μm” and “Below 50 μm” compartments of the grafted cortex. Synapsin expression did not significantly vary between compartments. However, Homer 1 density was lower than Synapsin density in the “Below 50 μm” and “Above 50 μm” compartments, but not in the mouse cortex compartment (Figure 3). Thus, there is a significant difference in density between the post- and pre-synaptic markers in the human cells, due to a significantly lower post-synaptic marker density, at 7 mpi. This difference is not significant anymore when the human cells have developed in close proximity to mouse cells.

In addition, the density of Homer1 puncta was significantly lower in the “Above 50 μm” compartment than in the “Below 50 μm” compartment which is closer to the mouse cortex. These data suggest that there is a difference in post-synaptic protein density in grafted human cells depending on their proximity to the mouse cells (Figure 4E ii).

### Human and mouse cells interact in the formation of the graft

**FIGURE 5:**
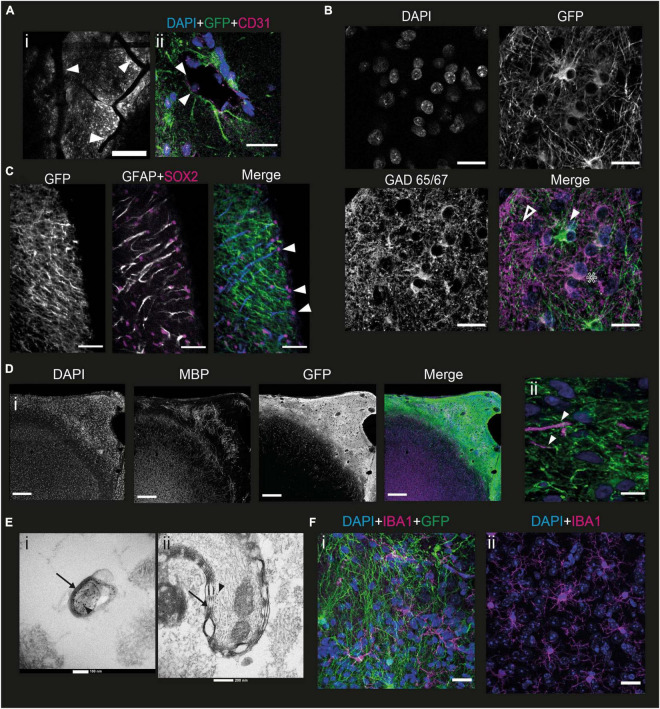
Mouse cells interact with the graft to form a functional cortex. **(A)** (i) Image of a mouse brain at 6 mpi taken under the 2P microscope showing grafted cells in grayscale with blood vessels going through the graft (white arrowheads). Scale bar = 200 μm. (ii) immunofluorescence staining of a mouse brain 6 mpi with antibodies directed against CD31, a marker for endothelial cells. Depicted is a putative blood vessel, lined with CD31-marked cells (white arrowheads). Scale bar = 20 μm. **(B)** Immunofluorescence staining of a mouse brain 6 mpi showing the expression of GFP, DAPI, and GAD65/67. The white arrowhead shows a human cell that does not express GAD65/67, the star shows a human cell expressing GAD65/67 while the hollow arrowhead shows a GAD65/67 positive cell that does not express GFP. Scale bar = 20 μm. **(C)** Immunofluorescence staining of a mouse brain at 1 mpi showing the expression of GFP, GFAP, and Sox2. The white arrowhead points to mouse radial glial cells, co-expressing all 3 markers. Scale bar = 50 μm. **(D)** Immunofluorescence staining of a mouse brain at 7 mpi showing expression of GFP, myelin basic protein (MBP), and DAPI (i) at the scale of the graft (scale bar = 200 μm) and (ii) at the scale of the cell (scale bar = 10 μm). White arrowheads point to human axons surrounded by MPB. **(E)** EM images taken from the sample at a depth of 140 μm (i) and 230 μm (ii) into the graft after immunohistochemistry against GFP with DAB precipitate. Black arrows point to myelin sheaths, black arrowheads point to DAB precipitates indicating human axons. **(F)** Immunofluorescence staining of a mouse brain at 6 mpi showing the expression of IBA1 (Magenta) and GFP (green), with DAPI (blue), in the grafted (i) and non-grafted (ii) parts of the cortex. Scale bar = 20 μm.

One of the main challenges with *in vitro* models of human cortical development, particularly minibrain organoids, is the potential lack of vascularization, which induces significant cell death within structures when they become too large, due to lack of oxygen (Chen et al., 2019). We therefore wanted to verify whether our grafting method allowed for the presence of blood vessels that would keep the developing neurons alive over the long term. The first indication was the presence of numerous blood vessels in the grafted part of the brain that could be observed under the 2P microscope (Figure 5A i). To elucidate this further, we stained brain slices at 6 mpi with antibodies directed against CD31, a protein of endothelial and hematopoietic cells (Van Mourik et al., 1985). Within the graft spaces are observed, as judged by fluorescence, which may contain nuclei of cells not expressing GFP. In addition, these nuclei are sometimes elongated. The edges of these openings express CD31 (Figure 5A ii). Thus, within the graft there are structures surrounded by endothelial cells that do not express GFP, therefore derived from the mouse, containing cells with elongated nuclei, suggesting that they are part of murine blood vessels.

To develop a functional neural network, the human pyramidal cells in the graft require interneurons. However, the embryonic origin of interneurons is different from the path of differentiation of projection cortical neurons in mice (Chu and Anderson, 2015). We wanted to verify the presence of interneurons in the graft and labeled slices of brain samples taken 6 mpi by immunofluorescence with antibodies directed against GFP and GAD65/67, an enzyme present in GABAergic neurons (Erlander et al., 1991). Punctiform labeling can be seen throughout the graft, corresponding to the projections of GABAergic neurons. Thus, there is innervation of the entire graft by GABAergic projections. This punctiform staining is rarely colocalized with the GFP staining, suggesting that a large part of this GABAergic innervation derives from mouse cells (Figure 5B).

In addition, staining can be observed on some cell bodies. Among these, cell bodies expressing GAD 65/67, but not GFP (Figure 5B, hollow arrowhead), can be identified. This suggests that these cells are mouse interneurons, or that they are human GABAergic neurons not expressing GCaMP6f. There are also neurons expressing both GFP and GAD65/67 (Figure 5B) indicating that some human cells express GABA. Finally, we were able to observe GFP-positive cell bodies not expressing GABA (Figure 5B, white arrowhead), consistent with our previous observation that most of the NPCs differentiate into upper layer cortical projection neurons.

To analyze the migration dynamics of murine astrocytes in the graft, we labeled the brains with an antibody directed against GFAP from 1 to 5 mpi. Astrocytes can be identified in high density in the graft from 1 mpi. These astrocytes are mainly mouse astrocytes since there is little colocalization between GFP and GFAP. In addition, at 1 mpi, mouse astrocytes exhibit an elongated morphology, and are organized in a ladder-like structure along the graft (Supplementary Figure 1). This phenotype does not correspond to that of mature astrocytes, and the organization is not unlike that of the radial glia, the precursor of neurons and astrocytes. It gradually disappears during the later stages of development studied (Supplementary Figure 1). Outside the graft, astrocytes also display a mature astrocytic phenotype. To validate this hypothesis, we labeled the 1 mpi slices with antibodies directed against GFAP, GFP, but also against Sox2, a multipotency marker expressed among others by radial glial cells (Ellis et al., 2004; Figure 5C). Mouse glial cells exhibit a Sox2 + nucleus. Many are found on the surface of the brain, but some are also found inside the graft. The extensions of the glial cells are all oriented in the same direction with respect to the nucleus. The mouse glial cells present in the graft 1 mpi therefore exhibit an organization with the soma toward the surface of the brain, and an extension toward the mouse brain. This supports the hypothesis that these cells are mouse radial glial cells, although we could not observe any human cells migrating along the glial extensions.

To function properly, human neurons require myelination from oligodendrocytes. However, the NPC-derived cells mainly differentiated into cortical neurons. In order to verify whether mouse cells could contribute to the myelination of human axons, we stained brains at 6 mpi for myelin basic protein (MBP), a protein present in myelin (Boggs, 2006). We could observe that mouse myelin was present in the graft. The myelin present in the graft was not organized radially like in the mouse cortex, but rather formed a seemingly random network, intermingled with the human cells. We could determine that it was not human because it never colocalized with the GFP staining (Figure 5D i). We could also identify some human axons, which appeared to be surrounded by mouse MBP (Figure 5D ii).

To confirm this finding, we performed electron microscopy (EM) on 7 mpi brains. As a means of validating that the myelinated axons that we observe would be human, we stained for GFP by immunohistochemistry, using peroxidase and DAB. DAB is both black in white light and dense to electrons, making it a useful tool to combine light and EM. We imaged samples at various depths from the surface, which corresponds to various depths into the graft, as the graft is mainly present superficially to layer I. We were able to observe human axons labeled with DAB wrapped in a myelin sheath, confirming that mouse oligodendrocytes do indeed myelinate human axons (Figure 5E).

Microglia play an important role during development (Reemst et al., 2016) and in the immunity of the central nervous system. In particular, they are involved in synapse pruning, glial healing, and inflammatory reactions in the brain. To verify whether the grafting of NPCs derived from hiPSCs had an impact on the morphology and behavior of murine microglia, we performed immunofluorescence labeling with antibodies directed against IBA1, a marker specific to microglia (Ito et al., 1998). Microglial cells can be observed in the graft (Figure 5F i). There is no colocalization between IBA1 and GFP, indicating that the microglia present in the graft are of murine origin. In addition, the morphology and density of the microglial cells present in the graft and in a cortical region of the mouse not containing the graft are similar (Figure 5F ii). We therefore conclude that the mouse microglia migrate into the graft but contact with the graft does not change their morphology or activation state in this NOD-SCID immunodeficient mouse line.

### Functional activity in the graft is that of a developing brain

The developing brain has been shown to display specific patterns of activity throughout the early stages of neuronal development in rodents (Crépel et al., 2007; Kilb et al., 2011) and humans (Eswaran et al., 2007; Moore et al., 2009; Moore et al., 2011). However, direct access to functional human developing neurons, while they go through these functional developmental stages, has been limited in available models so far. Our model therefore provides the opportunity to observe the evolution of calcium activity at the cellular level in a human developing neuron.

**FIGURE 6:**
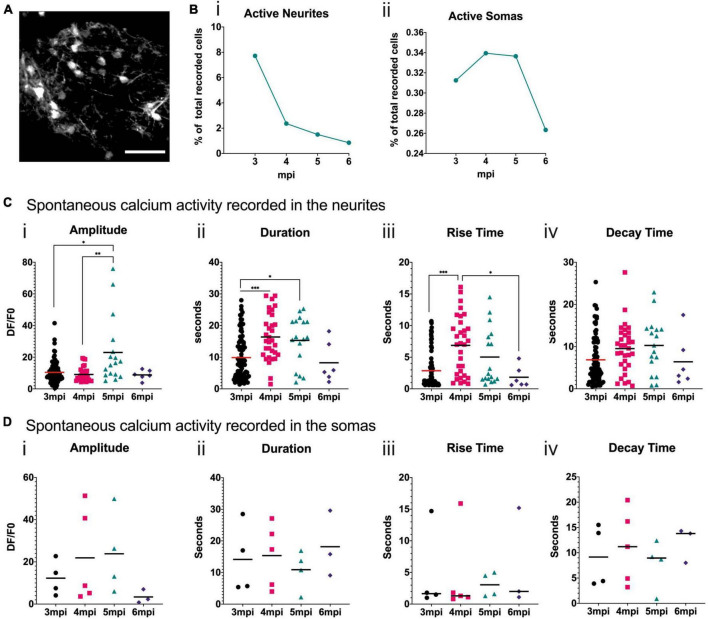
Spontaneous calcium activity in the grafted neurons reflects a developing brain. **(A)** Representative field of view of a mouse cortex observed at 6 mpi under the 2P microscope. GCaMP6f fluorescence is in grayscale. Scale bar = 50 μm. **(B)** Proportion of neurite (i) or soma (ii) ROIs where spontaneous activity was recorded for each time point. **(C,D)** Quantification of calcium transient amplitude (i), duration (ii), rise-time (iii) and decay-time (iv) for each time point in the neurite **(C)** or soma **(D)** ROI. Each symbol in the graph corresponds to one calcium transient, horizontal bars represent the mean for each time point. Statistical test: Kruskal–Wallis test with multiple comparison. *p < 0.05, **p < 0.01, ***p < 0.001.

To analyze the neuronal activity patterns of the developing grafted human cells, we performed surgery 2 mpi to implant cranial windows. Cells were visible through the cranial window and the spontaneous calcium activity in awake head-fixed mice was then recorded at 3, 4, 5, and 6 mpi. Figure 6A shows a representative field of view taken under the 2P microscope. We focused on the neurons closest to the surface of the brain. For each field of view, we measured fluorescence variations in manually defined regions of interest (ROIs) corresponding to cell somas or cell processes, which we refer to as neurites. We calculated the proportion of ROIs that were active during the recording times for each time point and both ROI types. We observed that the proportion of ROIs with spontaneous calcium activity is higher for neurites than for somas. For both ROI types, this activity decreased with time (Figure 6B). For the soma ROI, we could never observe more than one calcium event in any 5 min movie, whereas for the neurites’ ROI, multiple events were often observed, especially at 3 mpi (Supplementary Figure 2).

In the neurites, the amplitude of the spontaneous calcium transients is significantly higher at 5 mpi than at 3 and 4 mpi (Figure 6C). The duration of the spontaneous calcium transients increases significantly between 3 and 4, and 4 and 5 mpi (Figure 6C). The rise time significantly increases between 3 and 4 mpi and then significantly decreases by 6 mpi (Figure 6C). This suggests a shift in calcium transient shape (Supplementary Figure 3), consistent with shifts observed in mouse developing cortex (Allène et al., 2008). In the somas, some trends can be observed but are not statistically significant due to the small number of active human cells (Figure 6D), that are very close to the surface of the brain.

### The ultrastructure of the graft is reminiscent of that of a pre-natal primate brain

For immunofluorescence experiments described above, the brains were perfused with low fixative concentrations. Such a limited fixation is detrimental for the preservation of the brain ultrastructural quality, as compared to standard procedures (Bourgeois et al., 1994). Despite this technical limitation, we investigated this human xenograft tissue using EM.

**FIGURE 7:**
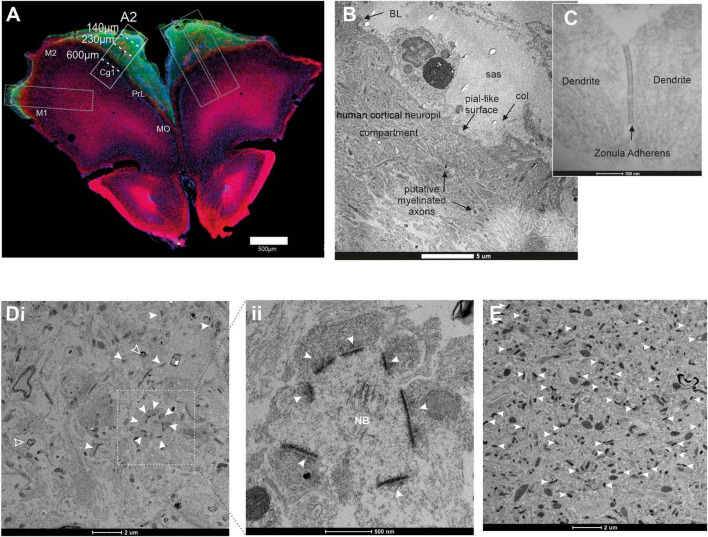
Observation of the structure of the graft at 7 mpi with EM. **(A)** Immunofluorescence staining with antibodies directed against GFP (green) to target human cells and MBP (magenta) to target myelin. The selected long vertical sample of human xenograft tissue (dashed rectangle A2) was dissected out from the section. It was then explored at the EM level, for a step-by-step descent (dashed lines) along a vertical probe from the putative pial surface of the human xenograft down to the putative host mouse cingulate cortical area (Cg1), at the depth of circa –600 μm. Scale bar = 500 μm. **(B)** EM image of the human xenograft taken near the surface of the vertical probe. Ultrastructures include a pial-like surface with basal lamina (BL), the subarachnoid space (Sas), containing bundles of collagen fibers (Col), and unidentified cells. Under the pial-like surface, the parenchyma of the human xenograft presents a loose neuropil compartment with non- artefactual large extracellular spaces. Some myelinated axons are also present (indicated with black arrows). Scale bar = 5 μm. **(C)** 20 μm below the surface of the xenograft we observed multiple zonula adherens (black arrow). **(D)** (i) 230 μm into the graft. Myelinated axons are present (hollow arrowheads). In this 258 μm^2^ field, 12 synaptic profiles can be observed (white arrowheads), each one confirmed at higher magnification as in panel **(D)** (ii). This gives an estimated raw areal density of 4 synaptic profiles/100 μm^2^ of neuropil. Scale bar = 2 μm. (ii) Magnification of what could be interpreted as a large dendritic shaft containing a Nissl Body (NB) surrounded by synaptic profiles (white arrowheads). **(E)** 600 μm from the surface of the graft. At this depth, a much higher density of synaptic profiles is observed (white arrowheads). This sample is now in the cerebral cortex of the host mouse at 7 mpi. The neuropil of the murine cortical tissue is mature. In this 258 μm^2^ field 53 synaptic profiles can be observed (yellow arrows), each one confirmed at higher magnification. This gives an estimated raw areal density of 20 synaptic profiles/100 μm^2^ of neuropil. Scale bar = 2 μm.

In the brain of the 7 mpi mouse, coronal vibratome sections (Figure 7) were prepared for parallel immunofluorescence and EM. The selected domain of the human xenograft displaying “human” GFP (hGFP; green), covers the fields of Motor (M2), Cingulate (Cg1), and Prelimbic (PrL) cortical areas of the host mouse, displaying “mouse” MBP (mMBP; red), located by immunofluorescence. A long vertical sample of tissue (corresponding to dashed rectangle A2 in Figure 7) was dissected out from a neighboring section, which underwent immunohistochemistry against GFP, with a DAB chromogen. It was then explored by EM in a step by step descent [along the thin horizontal white line shown in the dashed rectangle (Figure 7)], from the pial surface (starting level 0) of the human xenograft, down to the host mouse cortical tissue, at the depth level of *circa* −600 μm.

Despite poor ultrastructural preservation at the surface of the human xenograft (level 0 of the vertical descent probe), one can observe a pial-like surface with basal lamina (BL), a subarachnoid space (Sas) containing bundles of collagen fibers (Col) and unidentified cells (putative macrophage or mastocyte). Under the pial surface, the parenchyma of the human xenograft presents a loose neuropil compartment with non-artefactual large extracellular spaces (S, stroma). *Zonula adherens* contacts between neurites were also observed (Figure 7C). Some myelinated axons are also present.

At a depth of *circa* −230 μm from the pial surface the electron micrograph (Figure 7D i) is about halfway down in the human xenograft. Myelinated axons are present (arrowheads). In this 258 μm^2^ field, 12 synaptic profiles can be observed (arrowheads), each one confirmed at higher magnification as in Figure 7D ii. This gives an estimated raw areal density of 4 synaptic profiles/100 μm^2^ of human xenograft tissue. At a higher magnification, in the same EM field (Figure 7D ii), 7 synaptic profiles are distributed around what could be interpreted as a large dendritic shaft containing a Nissl Body (NB) (Scale bar = 500 nm). These NBs are aggregates of rough endoplasmic reticulum involved in intense protein synthetic activity close to these synaptic profiles [see in Peters et al. (1991), page 85, their Figures 3–6].

The electron micrograph presented in Figure 7E is taken at a depth of *circa*−600 μm from the pial surface. This sample is now in the cerebral cortex of the host mouse. In the 258 μm^2^ field presented here, 53 synaptic profiles can be observed (arrows). This gives an estimated raw areal density of 20 synaptic profiles/100 μm^2^. This density per unit area of murine cortical tissue is similar to the areal density of 25 synaptic profiles/100 μm^2^ reported by Schuz and Palm (1989) in the cerebral cortex of adult mice. This strongly suggests that here, too, the tempo of maturation of the murine cerebral cortex is not significantly altered by the presence of the human xenograft.

## Discussion

We describe here the long-term development, anatomical, morphological, and functional features of transplanted human iPSC-derived pyramidal cells in the mouse cortex over 7 months. Starting from single-cell sequenced NPCs with a high degree of transcriptional homogeneity, we followed their intrinsic neotenic behavior, and their graft-versus-host consequences. Our technique is inspired by previous work using hESC-derived pyramidal cells (Espuny-Camacho et al., 2013; Linaro et al., 2019) and more recently the more versatile hiPSC (Real et al., 2018). These and other studies have revealed several conserved features between these two sources. hiPSCs offer the advantage of an ethically more acceptable origin from living people and allow to correlate clinical parameters with cellular phenotypes of a given genome, including the CRISPR-Cas9 mediated isogenic manipulation. A further recent development is the direct transplantation of hiPSC-derived cortical organoids into rat recipients (Revah et al., 2022). This allows to combine the advantages of an initial culture period with long-term studies *in vivo*. Under the conditions of extensive graft integration, a change in rat behavior was observed, making this potentially the method of choice for the analysis of graft versus host action (Vermaercke et al., 2022).

Our work occupies a middle ground. As exemplified in Figure 7, large parts of the graft are positioned “on top” of the mouse cortex, almost the equivalent of an “organoid-like” structure developing *in vivo* during the 7 month follow-up period, and fully vascularized. The properties of the graft can thus be analyzed as a function of the distance from the mouse neurons. This has been demonstrated to be an important parameter in our fluorescent synaptic marker and EM studies. However, we have only used one single line, and confirmation will be carried out in follow-up experiments.

### Neoteny in transplanted human iPSC-derived neurons

As described before by us and others for transplanted hiPSCs (Shi et al., 2012; D’Alessio et al., 2020), the transplanted NPCs mature over time into pyramidal neurons. Using established markers like Cux1, their fate corresponds largely to upper cortical layer pyramidal neurons. A protocol similar to the one we used has previously been shown to favor differentiation toward upper layer cortical neurons *in vitro*, by increasing the number of cell divisions at the precursor stage. We implanted the neural precursors at passage 10–12 in order to amplify and bank them, reaching a similar number of passages as in this paper (Boissart et al., 2013).

Only a small number of interneurons and graft-derived human astrocytes could be identified in line with the chosen differentiation culture protocol (Gaspard et al., 2008; van den Ameele et al., 2014). The grafts form mostly little “clusters” in the mouse brain, but our quantification of mouse-versus-human neurons shows that up to 15% of mouse neurons are present inside the xenograft. This provides direct contact between the two classes of neurons.

We have followed the increasing average spine density of the human pyramidal cells using the recently developed DIVA system (El-Beheiry et al., 2020). Whereas no dendritic spines were detectable at 1 mpi, the average density was 0.12 per μm at 6 mpi. This is consistent with what has been reported by others for human cells developing 5–6 months in the mouse brain (Linaro et al., 2019).

We have then carried out a comprehensive analysis of synaptic marker profiles in these two populations using Synapsin and Homer1 as pre- and postsynaptic labels, respectively. Whereas in mouse-only cortical areas these two densities correspond perfectly, there is a markedly lower density of Homer1 present in human neurons. This indicates a “delayed” post-synaptic maturation of the human neurons, whereas pre-synaptic marker densities are the same, indicating innervation from mouse neurons onto human neurons. We have not further analyzed the interspecies distribution of the two markers. The inhibitory marker GAD65/67 is widely expressed within the human graft, with an almost exclusive origin from mouse interneurons, see Figure 3B. Therefore, inhibitory input is also likely present at that point in post-natal development, although the precise E/I balance needs to still be established using slice electrophysiology.

### The grafted human neurons induce transient developmental features in the mouse brain

We have also underlined in this study the reciprocal influence that the mouse and human cells have during the development of the grafted hiPSCs. We have shown that while the murine glial cells are fully mature in the contralateral cortex to the graft, the murine glial cells around the graft exhibit a radial glial cell phenotype. Moreover, we have shown here for the first time that mouse oligodendrocytes myelinate human axons at 7 mpi.

These interactions can be explained by the timing of the injection. Indeed, at P1, the stage at which the human cells are implanted into the mouse cortex, mouse oligodendrogenesis and astrogenesis are still ongoing (Reemst et al., 2016), leaving a window of opportunity for potential developmental signals coming from the human cells to disrupt, or slow down, these stages of development.

We also observe the presence of microglia in the graft, but their density and morphology are similar to those observed in the contralateral part of the mouse cortex, suggesting a limited impact of the presence of the human cells on microglial development.

### The grafted human neurons are functional in the mouse brain and change their activity over time

A highlight of the present study is the comprehensive analysis of functional maturation over time using *in vivo* chronic 2P imaging in the awake mouse. We identify a striking difference in the activity patterns of dendrites vs. soma during development. Whereas up to 8% of dendrites exhibit detectable calcium transients, this remains on the order of 0.35% for the soma, in individual recordings. Over time, and at 6 mpi, these two activity patterns correspond and indicate a percentage of 1 in 100 active neurons during our recordings lasting 5 or 10 min.

This increase in dendritic activity patterns compared to the soma potentially reflects important differences between human and rodent neurons. In a study analyzing dendritic compartmentalization of adult rat and human layer 5 neurons in slices (Beaulieu-Laroche et al., 2018), the authors found that human cortical neurons exhibit a higher degree of voltage compartmentalization compared to rodent counterparts due to lower ion channel densities across larger dendritic surfaces. Compared to rat dendrites, distal human dendrites provide limited excitation to the soma, even in the presence of dendritic spikes. Human somas also exhibit less bursting due to reduced recruitment of dendritic electrogenesis. Similar studies on human fetal neurons have not yet been carried out, but our present work points to a potential mechanism underlying the observed activity patterns in the developing human dendrites, that do not lead to the generation of calcium transients/spiking in the soma. In a “conflicting” study in human slice recordings from layer 2/3 pyramidal neurons, different authors observed a remarkably lower specific membrane capacitance of human L2/3. This level of sophisticated analysis can now be reached in our present experimental model, due to its reproducibility, and the possibility to genetically modify the hiPSCs before transplantation.

This small number of detectably “active” neurons might also reflect the heterogeneity of the differentiation within the graft. Indeed, as our results show, at 6 mpi maturing neurons and pluripotent cells coexist within the graft. It could also be related to the percentage of NPCs expressing detectably GCaMP6f. Moreover, our observations based on the EM study of the graft lead us to believe that the graft cells located fully within the mouse brain parenchyma might be further along in their differentiation process than the human cells at the surface. However, our cranial windows only enable us to record cells located within 200 μm of the surface, where the neurons might be less mature and therefore display slower and less frequent fluctuations.

The calcium transients were analyzed in more detail for both the dendritic and somatic compartments. Using advanced image processing, the amplitude, duration, rise-, and decay-times were quantified. For the dendrites, the amplitudes are fairly homogeneous at 3, 4, and 6 mpi with an average around 10 for ΔF/F, whereas a marked increase is observed at 5 mpi. More important differences are evident in the duration, with a significant increase at 4 and 5 mpi. This increase in duration is due to an increased rise-time at those ages, whereas there is no statistically significant difference in decay-time. Whereas rise-times are on the order of 5 s, decay-times are about twice as long. These are very slow time scales compared to mature neurons. It is reminiscent of descriptions focusing on embryonic brain activity from rodent literature (Allène et al., 2008; Allene and Cossart, 2010), and the extant literature on work in human fetal brain slices (Khazipov and Luhmann, 2006; Allene and Cossart, 2010). No significant differences have been observed in all these parameters for somatic activity, for the four time points, possibly due to the small number of events recorded. A further limitation is the extent of GCaMP6f expression pattern. When analyzing the expression level of the NPCs before transplantation, this is on the order of 3.7%. But it allows the GCaMP6F-positive neurons to be well separated, and permits the simultaneous analysis of soma and neurites, easily discernible.

### The grafted human neurons exhibit ultra-structural features of fetal development

Despite the poor preservation of its ultrastructural quality, we examined a sample of the GFP + human xenograft tissue at 7 mpi by EM. At the upper surface of the graft, we could observe an immature pial-like surface with a faint basal lamina and a subarachnoid space containing a few bundles of collagen fibers. The parenchyma of the xenograft presented an immature neuropil with large extracellular spaces hosting rare myelinated axons. The presence of DAB precipitates in the axoplasms of myelinated axons, after immunolabeling of GFP, suggests that they are human axons. The absence of DAB precipitates within the myelin sheath is consistent with myelination of human axons by murine oligodendrocytes as shown using immunofluorescence images.

At the EM level, our observation of an immature neuropil along with large extracellular spaces in the human xenograft, is reminiscent of pre-natal early fetal non-human primates. As a matter of fact, we previously observed similar ultrastructural features in prefrontal cerebral cortex of *Macaca rhesus* (Bourgeois et al., 1994) (see their Figure 3), as well as in several other cortical areas [see additional references in Bourgeois (2010)]. These ultra-structural features have also been observed at the EM in human early fetal cerebral cortex (Molliver et al., 1973) (their Figures 1–7), and (Zecevic, 1998) (their Figures 2–3). Such observations are also commonly described in fetal cerebral cortex of other mammalian species investigated at the EM, for example the cerebral cortex of rat (Blue and Parnavelas, 1983) (their Figures 1–12), to quote just one.

It is important to underline here that these immature ultra-structural features also coincide with the early onset of synaptogenesis, either during gestation in non-human and human primates, and at birth in rodents. Our observation of a very low density of synaptic profiles in our 7 months-old human xenograft, is coherent with the observations reported in the fetal human cerebral cortex (Huttenlocher and Dabholkar, 1997).

The sample at the depth of *circa* −230 μm from the pial surface is in the middle of the human xenograft. Myelinated axons are present. In the 258 μm^2^ field illustrated, 12 synaptic profiles could be observed, giving a raw areal density of 4 synaptic profiles/100 μm^2^ of human xenograft. Such a low areal density is comparable to the low areal density of 3 synaptic profiles/100 μm^2^ reported by Huttenlocher and Dabholkar (1997) (see raw data in their Table 2), also using EM, in human prefrontal cortex at the end of gestation. The present observations suggest that the human xenograft after 7 months in the murine host has maintained the developmental tempo of synaptogenesis observed in human prefrontal cortex, *in utero*.

A sample at the depth level of *circa* −600 μm from the pial surface is now in the cerebral cortex of the mouse host. In the 258 μm^2^ field observed here, 53 synaptic profiles can be observed, which gives a raw areal density of 20 synaptic profiles/100 μm^2^. This density per unit area of murine cortical tissue is quite similar to the areal density of 25 synaptic profiles/100 μm^2^ reported by Schuz and Palm (1989) (see raw data in their Table 1), who were also using EM in the prefrontal cortex (area 8) of adult mice. This strongly suggests that here, too, the tempo of synaptogenesis in the murine cerebral cortex has not been significantly altered by the presence of the human xenograft during 7 months.

The present qualitative EM ultrastructural description also validates our present experimental mouse model in 3 points: First, our description of synaptic profiles using EM in the human xenograft is the first extensive one in a mouse model. Second, it confirms, at the ultrastructural level, the immunofluorescence demonstration of synaptic contacts inside the human xenograft. *Third*, it suggests that the *tempo* of synaptogenesis in the human xenograft follows a cell-centered developmental program, and does not alter the mouse own cortical developmental program. This time-consuming qualitative observation at the electron microscope, using vertical probe, opens to less time-consuming statistical quantitative EM studies, previously validated (Granger et al., 1995), in the future experimental investigations.

The presence of an immature neuropil and a low density of synapses, along with functional imaging of spontaneous neuronal activity, suggest that 7 months after its seeding, the human xenograft displays the functional and structural maturity of a late fetal human brain. All these preliminary observations on human xenografts are consistent with the protracted development of human neurons observed *in vitro* (Gaspard et al., 2008).

## Materials and methods

### Cells

The origin and reprogramming of the iPS cell line used in this paper were described in Lemonnier et al. (2011). Briefly, cultured fibroblasts were exposed to retroviral vectors encoding OCT4, SOX2, KLF4 combined with a vector coding for c-MYC. After exposure, fibroblasts were grown on a feeder layer of irradiated mouse embryonic fibroblasts in the presence of basic fibroblast growth factor (FGF2, 10 ng/ml). The cells were propagated for at least 20 passages on iMEF, and characterized as pluripotent stem cells, after being controlled for genomic integrity.

### hiPSC differentiation

#### Day (D) 0: formation of embryoid bodies

When iPS cells cultured in 6 cm diameter Petri dishes reach 60/70% confluence the medium is aspirated and the cells are incubated for 2 min with 1 ml 0.5 mM of EDTA (Invitrogen; 155750) at 37°C to facilitate cell detachment. EDTA is then replaced with 1 mL of E8 medium (STEMCELL technologies TeSR™-E8™ Kit for hESC/hiPSC Maintenance). Then, the detached iPS cells are collected and transferred to a 15 mL Falcon.

Cell clusters are left to sink to the bottom of the tube for 10 min, then the pellet is resuspended in 10 mL of NMB medium.

NMB Medium:

(1) 25 ml F12 (ThermoFisher; 11524436)
(2) 25 ml Neurobasal (ThermoFisher; 11570556)
(3) 0.5 ml Penicillin/Streptomycin (11548876 ThermoFisher)
(4) 0.5 ml N2 (17502001 ThermoFisher)
(5) 1 ml B27 without vitamin A (11500446 ThermoFisher)
(6) 50 μl β-Mercaptoethanol 50 mM (31350010 ThermoFisher)
(7) Filter at 0.22 μm

Supplemented with:

(1) 10 μM SB431542 (130-105-336 Miltenyi)
(2) 100 nM LDN193189 (130-106-540 Miltenyi)
(3) 10 μM Y27632 (130-103-922 StemMACS) on the first day

The cells are transferred to a T25 flask and the medium is changed every day for 1 week.

#### D7: recovering the embryoid bodies

The embryoid bodies are allowed to sink to the bottom of the flask and the medium is aspirated and replaced with NPC medium supplemented with 20 ng/mL bFGF (130-093-842 Miltenyi) and 20 ng/mL EGF (130-093-825 Miltenyi) cell proliferation factors. Embryoid bodies are then cultured in Petri dishes with a coating of Polyornithine at 100 μg/ml (P8638 Sigma)/Laminin at 20 ng/ml (L2020 Sigma) until formation of Rosettes.

NPC medium

(1) 500 mL Neurobasal
(2) 5 mL Penicillin/Streptomycin
(3) 10 mL B27 without vitamin A
(4) 5 mL Glutamax (11574466 Gibco)
(5) Filtered at 0.22 μm

#### D12-14: rosettes selection and plating at P0

After washing with DMEM/F12 medium (ThermoFisher; 11524436), the medium is replaced by Neural Rosette Selection Reagent (STEMCELL technologies 05832) and incubated for 15 min. The Rosettes are then mechanically detached and centrifuged at 350 *g* for 5 min. The pellet is then resuspended in NPC medium supplemented with bFGF (20 ng/mL) and EGF (20 ng/mL) then seeded on a 6 cm diameter Petri dish.

#### D13-17 first passages of NPC

The NPC medium is changed every day until the seeded precursors reach 80–90% confluence. At this point, the NPCs undergo their first passage. During the first 3 passages, the NPC medium is supplemented with the Rock inhibitor Y27632 10 μM to decrease apoptosis related to cell dissociation.

Cells are detached with Trypsin and clusters are mechanically dissociated with a P1000 pipette. The dissociated cells are centrifuged at 1,500 rpm for 5 min. Then the pellet is resuspended in 1 mL of Neurobasal medium per dish and cells are counted on a Malassez cell. The neural precursors are then seeded on a new Petri dish at 93,000 cells/cm2. The NPCs are maintained on a Geltrex coating at 300 μg/mL (12063569 Gibco).

### For injections

Right before the injection, a confluent petri dish of NPCs is detached in trypsin for 5 min at 37°C. The dissociated cells were then centrifuged in NPC medium at 1,500 rpm for 5 min. The dissociated cells are then resuspended in NPC medium and counted on a Malassez cell. The cells are then centrifuged at 1,500 rpm for 5 min again and resuspended in PBS at a concentration of 200,000 cells/μL.

#### Generation of the lentiviral vector

To obtain a lentiviral vector expressing GCaMP6f, a plasmid construct coding for the GCaMP6f available on AddGene was used (Douglas Kim and GENIE Project, Addgene plasmid # 40755^1^ ; RRID:Addgene_40755), from which the GCaMP6f gene was extracted and inserted into a lentivirus backbone used previously in the laboratory (Morel et al., 2014). The GCaMP6f sequence was amplified from the pGP-CMV-GCaMP6f plasmid by PCR and an *Xho*I site was added at the 5′ end of the sequence as a floating tail (in lower case below).

Primer for: ctcgagGACTCAGATCTCGCCACCATG
Primer rev: CCTCTCTACAAATGTGGTATGGTATGGCTG

We chose to extract the GCaMP6f sequence from the starting plasmid by PCR and to subclone it into an intermediate vector by T/A cloning technique, which allowed us to perform mutagenesis add the *Spe*I site in 3′. To insert an *Xho*I restriction site in 5′ of the sequence, we created primers to amplify the sequence and added a floating tail on the primer in 5′ with the *Xho*I sequence (in lower case below).

Primer for: *ctcgag*GACTCAGATCTCGCCACCATG
Primer rev: CCTCTCTACAAATGTGGTATGGTATGGCTG

The amplified and extracted fragment is then inserted into a pGEMT plasmid (T/A cloning technique). To facilitate the extraction of the GCaMP6f sequence from the pGEMT plasmid, we introduced a mutation at the *Xho*I site within the GCaMP6f sequence by directed mutagenesis (lowercase below).

Mut-*Xho*I-For primer: GTCGGCTGAGCTCACT*t*GAGAA CGTCTATC
Mut-*Xho*I-Rev primer: GATATAGACGTTCTC*a*AGTGAG CTCGAC

For clones with the expected digestion profile, sequencing of the GCaMP6f containing zone was performed using primers based on the Sp6 and T7 sequences proposed by the sequencing company Eurofins. A clone was selected from those that had the GCaMP6f sequence in the correct direction and had the mutation in the *Xho*I restriction site sequence. The GCaMP6f fragment was then inserted into the CMV lentivirus backbone using a 3-strand ligation strategy, taking advantage of the *Xho*I, *Bss*HII and *Spe*I restriction sites. The final construction was fully verified by sequencing from the following primers distributed in both the sequences coding for lentiviral genes and our gene of interest:

Primer in the RRE sequence: CAACTCACAGTCTGGGCATC
CMV-for primer proposed by the sequencing company
beginning of the GCaMP6f sequence: GACCAC ATGAAGCACG
WPRE sequence: GTGGCCAGGATCCGATAATC
All selected clones contain the GCaMP6f sequence and all viral sequences.

### Animals

NOD.CB17-*Prkdc^scid^*/NCrCrl mice from Charles River (RRID:IMSR_CRL:394) were used for this study. Immunodeficient mice were necessary to avoid rejection of the cell graft. Experiments were carried out on mice of either sex. The experiments described in the present work were conducted in accordance with the guidelines on the ethical use of animals from the European Community Council Directive of 24 November 1986 (86/609/EEC) and in accordance with institutional animal welfare guidelines and were approved by Animalerie Centrale, Médecine du Travail and the Ethics Committee CETEA of Institut Pasteur, protocol number 170004.

Breeding cages of 1 male and 2 females were set up and monitored daily. If both females exhibited signs of gestation one of them was isolated to limit overcrowding in the cages. Otherwise, the trios were kept together as much as possible, to avoid male aggressiveness toward unfamiliar pups and isolation of the females. Animals were bred between the ages of 8 weeks to 5 months and litter size ranged from 6 to 11 pups.

#### Single cell sequencing of NPCs

Neural Precursor Cells were passaged as for injection but were diluted to 1,000 cells/μL in Neurobasal medium. 20 000 cells were loaded onto the Chromium Next GEM Chip G of the Chromium Next GEM Single Cell 3′ Reagent Kits v3.1 from 10X Genomics and a library was created according to the manufacturer’s protocol. The library was then sequenced with a NextSeq 500/550 High Output Kit v2.5 from Illumina. Cellranger (v.4.0.0) was used with refdata-gex-GRCh38-2020-A from Illumina as a reference with standard parameters to obtain a count matrix.

Of the 20 000 cells that were originally deposited for the first barcoding step, 5411 cells were identified after the sequencing. Expression values were transformed using log(+1) and normalized to equal pseudo counts. Seurat’s (version 4.3.0) (Hao et al., 2021) RunPCA with 15 components and 200 variable genes, using centering and scaling was followed Seurat’s clustering function (15 PCAs, resolution = 0.5, K = 10) to produce 8 clusters that were manually renamed (0–5.8 = NPC, cluster 6 = mature). Cells expressing at least one UMI of either NANOG or POU5F1 were assigned “stem-like.” All analyses and visualizations were performed using SCHNAPPs. A docker version of SCHNAPPs with the data is available on docker-hub (schnapps.Rais). Raw sequences and scripts to produce input data for SCHNAPPs and figures are available upon request.

#### NPC injections into newborn mouse pups

Injections were carried out at P1 (1 day after birth) in general, but some injections also at P0 or P2 due to cell confluence constraints and/or uncertainties on the date of birth.

The pups were collected one by one without disrupting the nest and put in a box lined with tissues. A 33 gauge Hamilton syringe with a stopper at 1 mm on the needle, was then loaded with 1 μL of cells. The pup was then delicately collected from the box and its head maintained with two fingers to show the bregma and lambda. This protocol targets the frontal cortex. The needle was then inserted at 1 mm laterally of the median line and 1 mm caudally of bregma. For functional imaging experiments, the injection was repeated on the other side. The pup was then placed back in the cage in the nest it was taken from. The mother was then monitored for a few minutes to make sure it would start caring for the pups again and the cage was put back in the ventilated rack. When they reached 3 weeks, the pups were weaned from the mother and kept in cages of 4 or 5 littermates.

### Histological analyses

Mice from each litter were perfused at 1 month post-injection (mpi), 2 mpi, 3 mpi, 4 mpi, 5 mpi, and 6 mpi. Fixation was carried out by transcardiac perfusion of anesthetized mice using 4% paraformaldehyde (PFA) and the brains were removed and post-fixed by immersion in PFA for 2 days at 4°C. A total of 50 μm sections of the whole brain were then performed using a vibratome, and the sections were preserved in PBS containing 0.2% sodium azide.

### Immunostainings

After permeabilization and blocking with 0.2% Triton X-100 and 5% horse serum in PBS the free-floating sections were incubated at 4°C overnight in primary antibodies. Appropriate secondary antibodies were used at room temperature for 3 h. For the nuclear staining, 4′,6-diamidino-2-phenylindole (DAPI) (600 ng/ml) was added 10 min before the end of the secondary antibody incubation. Details about antibodies used can be found in Supplementary Tables 1, 2.

### Chronic cranial windows

Mice were pre-medicated with buprenorphine (Buprecare) subcutaneously and anesthetized with isoflurane. The surgery was performed with the mouse in a stereotaxic frame. Xylocaine was applied subcutaneously under the scalp and on the skin before the skull was exposed. Mice were kept on a thermal blanket, and their eyes were protected with artificial tear ointment. The scalp was washed with three alternating swabs of 70% ethanol and betadine. Surgical tools were sterilized using a hot glass-bead sterilizer. A chronic cranial window was prepared as previously described (Koukouli et al., 2017). Briefly, the skull was gently thinned using a dental drill around the region of interest, and the thinned bone was removed using forceps, leaving the dura intact. It was then replaced with a 5 mm round glass slide and fixed to the skull with dental cement.

### Mouse handling for awake imaging

Mice were habituated to the imaging environment by handling and training for 2 weeks, as described previously (Koukouli et al., 2017). Briefly, the animals were habituated to the following conditions sequentially: (1) gentle handling, (2) free exploration of a 50-ml open-ended support tube (for the mouse to rest in during imaging), (3) incremental periods of mild head fixation in which the metal bar on the mouse’s head was briefly held in two fingers (2 s to 30 s) and (4) extended periods of head fixation on the mouse stage while the mouse rested in the support tube (5 min to 40 min). The same animals were imaged 3 days in a row for habituation purposes.

### *In vivo* two-photon imaging

*In vivo* imaging was performed with an Ultima IV two-photon laser-scanning microscope system (Bruker) using a 16 × , 0.8 N.A. water immersion objective (Nikon) with the femtosecond laser (Mai Tai DeepSee, Spectra Physics) tuned to 950 nm for imaging of cells expressing GCaMP6f.

Time-series movies of grafted cells populations expressing GCaMP6f were acquired at the frame rate of 30.33 Hz (405 × 405 μm field of view; 0.79 μm/pixel). The duration of each focal plane movie was 165 s (5,000 frames) for all time points in a first group of 5 mice and then extended to 330 s (10,000 frames) for all time points for all subsequent mouse groups to maximize the chances of observing somatic transients. Each mouse was recorded 3 days in a row for sessions of up to 30 min. On each recording day, 3 movies were recorded of non-overlapping fields of view within the grafted region.

### 2P data analysis

Image analysis was performed offline with ImageJ software. Images were stabilized using the “moco” software (Dubbs et al., 2016). Then, a mean-normalized maximum intensity projection of each movie was obtained using Fiji. This biased detection toward active cells. ROIs were manually selected using the ROI manager in Fiji, and the mean intensity of fluorescence was measured for each ROI using the multi measure tool. Detection of Ca^2+^ transients of individual neurons was performed automatically by using a custom-written toolbox in MATLAB (v2018b, The MathWorks, USA, RRID:SCR_001622).

Calcium traces obtained from 2P microscope movies were also analyzed with MATLAB. Typically, 50 calcium traces are generated from a film, each corresponding to the calcium signal of a single cell followed for about 5 min. These traces typically have 0 to 2 prominent peaks due to cell activation, with a rapid onset and a slower descent rate extending over several seconds.

Occasionally, they are mixed with other peaks, typically 10 to 50, equally large and long, due to the animal movements during acquisition. These artefactual peaks occur at the same time for all cells in the same visual field and can be of positive or negative amplitude, which can be exploited to filter them out of the analysis. First, all the cellular traces of a film are loaded into MATLAB. Each of them reports the fluorescence of the calcium reporter intensity in a single cell over time with an image interval of 32 ms. For each trace, we calculate the end signal F/F0:


dF/F0=(F⁢(t)-F0⁢(t))/F0⁢(t)

With:

– F(t) the raw fluorescence signal;
– F0(t) is the average of the raw fluorescence in the 50% lowest intensity over a sliding window of 30 s before the time t.

The dF/F0 signal is then filtered by a moving average over 0.8 s. We detect peaks in the resulting curve keeping only those peaks that have:

– a width greater than 0.8 s;
– an amplitude greater than a threshold given by the standard deviation of the measurement curve divided by a sensitivity factor 1;
– a prominence greater than the same threshold.

Each peak is inspected separately. First, we calculate AVGo and STDo, respectively the average and standard deviation of the absolute signal strength in all other cell traces at the time of the peak. If the peak intensity is less than


(AVGo+STDo)/Sensitivity⁢2

then the peak is considered false and removed from the analysis. The dynamics of the remaining peaks are then analyzed, and the rise-time is measured as the time it takes to move from 10% of the peak value to the peak, and the decay-time is measured as the time it takes for the peak to decay to 10% of its value.

The methodology detailed above depends on two sensitivity parameters sensitivity1 (*de facto* sensitivity to detect all peaks) and sensitivity2 (sensitivity factor to reject artifactual peaks).

They were manually adjusted on a sample of about 5 videos to values of 0.2 and 0.15, respectively, and then used for all the films in this study.

### Quantifications

#### Sox2 and STEM101 quantification

The quantification of Sox2 and Stem101 expression was performed using the ImageJ (RRID:SCR_003070). Slices were imaged with an Inverted widefield Microscope (Zeiss) equipped with an apotome grid. A tilescan containing all the visible Stem101 positive cells (graft) was imaged at 20 different points in depth, with a step of 0.5 μm resulting in a slice of 10 μm depth for each brain slice. Up to 3 slices per brain were imaged when the graft was large enough. Those images were then processed using a custom-written ImageJ macro. First, images were averaged 4 by 4 in the z direction, to obtain 5 z points for each slice. An ROI corresponding to the graft (including all the Stem101 positive somas above layer 1 of the mouse cortex), was drawn for the first and last z point, to define the Graft ROI. The other 3 graft ROIs were interpolated based on the extreme points. For each z point, the graft ROI was then divided in two separate ROIs, one from the deeper limit of the Graft ROI to 50 μm into the graft called Below 50 μm and one above the 50 μm limit called Above 50 μm. All tissues not included into the Graft ROI were considered part of the Mouse Cortex ROI. Within all ROIs, each channel was thresholded and converted to a mask. The DAPI staining was used as a control of the background signal and all detected fluorescence not overlapping with the DAPI signal were considered background and removed. Intersection ROI were then created to assess colocalization between the different channels. The area of these intersection masks were then used as a proxy for the number of cells. The area of each intersection for each z point was then normalized to the total DAPI area and all 5 z points were averaged together. For each region, the results are expressed in percentage of surface of GFP cells also expressing Sox2, to account for differences in the total surface imaged.

#### Synaptic marker quantification

Slices were imaged on a ZEISS LSM 700 inverted confocal microscope. For each brain, 3 slices were imaged (when the graft was large enough) corresponding to the most rostral, thickest, and more caudal part of the graft. For each slice, 3 images were taken. Images were tile scans from the surface of the brain to the deepest region of the brain containing human projections and z-stacks spanning 5 μm separated by 1 μm depth. The quantification of synaptic marker expression was performed using the Icy software (De Chaumont et al., 2012). Regions of Interest were defined as follows: The graft region is defined as the region which includes all the GFP-positive somas. This ROI is then reduced by 50 μm is then split into 2 regions: the Below 50 μm ROI corresponds to the tissue between the 50 μm of graft closest to the mouse cortex, while the region closest to the brain surface is the Above 50 μm ROI. The mouse cortex ROI includes all the tissue not containing GFP positive somas, it does include the human projections into the mouse cortex. The human cells ROI corresponds to the green channel with a threshold of 5,000. Each ROI was then intersected with the Human Cells ROI to specifically count the spots present on the human cells for each region. For each of these ROIs, the spot detector program was run for both the Synapsin and the Homer1 channel. The density of points for each region corresponds to the number of points detected divided by the surface of ROI intersected with the Human cells channel.

### Dendrites and spine tracing

For each mouse, brain slices stained with anti-GFP antibodies were first imaged at low resolution to identify regions where isolated cells were visible within the graft. Then a selection of up to 5 slices per mouse was imaged with a confocal microscope (Leica SP8) at 63X magnification. Z-stacks with a step of 0.3 μm containing the entire depth of the slice stained with GFP were taken to follow the dendrites as far as possible. The images were then treated in Fiji (image J) with a Gaussian blur filter to remove some background noise. The images were then uploaded in the DIVA virtual reality software (El-Beheiry et al., 2020). In the software, pyramidal neurons were identified based on morphology. Primary dendrites were traced using the Ruler tool. We focused on dendrites that remained within the plane of the section and were clearly differentiated from neighboring projections throughout the analyzed image. Then, for each traced dendrite the corresponding dendritic spines’ position was recorded using the Counter tool. They were identified visually from their thin diameter (less than 1 μm), short length (less than 2 μm), and presence of a larger head compared to the neck. This is in line with Benavides-Piccione et al. (2013).

### Statistical analysis

The statistical significance of the impact of the region (mouse cortex, graft below 50 μm, graft above 50 μm) and time point on the expression of the Sox2 marker (Figure 3D) was assessed with a Two-way ANOVA, followed by A *post hoc* Dunn multiple comparison *p < 0.05, **p < 0.005, *p < 0.001 (*n* = 18 mice).

The statistical difference in average spine density in spines per μm of dendrite at 1, 3, and 6 mpi was assessed with a Kruskal–Wallis multiple comparison test. **p < 0.01 (Figure 4E i) Quantification.

The statistical difference in density of puncta per μm2 for Synapsin and Homer1 stainings, in human cells expressing GFP contained in defined compartments of the mouse cortex (Figure 4E ii) was assessed by a Wilcoxon test, *p < 0.05, ns = not significant.

Statistical differences in the quantification of calcium transient amplitude, duration, rise time and decay time for each time point in the neurites (Figure 6C) or soma (Figure 6D) ROI was assessed by a Kruskal–Wallis test with multiple comparison. *p < 0.05, **p < 0.01, ***p < 0.001.

### Electron microscopy

#### Tissue sampling

The 7-month-old mice were deeply anesthetized with a mix of 75 mg/kg of Ketamine (IMALGENE 1000) and 10 mg/kg of Xylazine (Rompun), followed by intra-ventricular perfusion of 50 mL of 0.2M HEPES buffer followed by 200 mL of ice cold PFA 4% (Electron Microscopy Sciences 15713S) in 0.2M HEPES buffer. The brains were collected and stored overnight in PFA 4% HEPES buffer for post-fixation in the cold room.

The brains were cut into 70 μm vibratome sections collected in ice cold PBS sequentially: one out of three sections was kept for electron microscopy, one out of three slices was kept for immunofluorescence, and the rest was stored at 4°C in 0.2% PBS Azide. The sections intended either for immunofluorescence or electron microscopy were treated in parallel using the free-floating sections immunohistochemistry protocol directed against GFP.

#### Immunohistochemistry for electron microscopy

The free aldehydes were neutralized by placing the free-floating sections in a 50 mM NH_4_Cl solution at 4°C for 30 min. The sections were then rinsed and the endogenous peroxidases were neutralized with a 3% H_2_O_2_ solution at 4°C for 20 min. Then, they were treated for 30’ at 4°C in a PBS solution containing 0.5% Triton X-100, 1% Bovine Serum Albumin (BSA), and 10% Normal Horse Serum (NHS) to saturate the non-specific binding sites.

The GFP proteins in the sections were immunolabeled with rabbit anti- (Thermo Fisher Scientific Cat# A-6455, RRID:AB_221570) diluted 1:750, in PBS with 2% NHS, 0.2% Triton overnight at 4°C in agitation.

After rinsing in PBS, the sections were incubated with the secondary antibody: biotinylated goat IgG directed against rabbit (Vector Laboratories Cat# BA-1000, RRID:AB_2313606), in a 2% NHS-containing solution 2 h at room temperature. After rinsing in PBS, the sections were incubated with Avidine peroxidase complex (ABC complex, Vectastain Elite PK 6100) for 45 min at room temperature. The sections were rinsed with PBS and then rinsed with 0.1M tris–HCl buffer.

Finally, the labeling was revealed by DAB: the sections were incubated in diaminobenzidine (DAB EASY 10 mg/tablet; Acros Organics, reference 7411-49-6) previously dissolved (10 mg in 50 mL) and shaken for 3 min. After 3 min a solution of H_2_O_2_ was then added for a final H_2_O_2_ concentration of 0.014%. The reaction was stopped after 1 min by adding a large volume of 0.1M Tris HCl buffer, the sections were rinsed 3 times in this buffer, and 3 times in a 0.1M cacodylate buffer pH 7.2.

At this stage, if the graft is large enough, it could be seen with the naked eye with the DAB marking. Protein complexes in the DAB-labeled sections were fixed for 1 h in 2% glutaraldehyde in cacodylate buffer (EM grade; Electron Microscopy Sciences), then rinsed in cacodylate buffer.

Under a binocular microscope, elongated rectangular samples of brain tissue spanning the full vertical depth of the graft were dissected out from the vibratome sections based on the immunofluorescence images obtained from alternative vibratome sections treated in parallel. The bottom of each vertical sample was marked by an oblique cut to keep track of its exact orientation through the next treatments.

#### Preparation of samples for electron microscopy

In these vertical samples of xenografts, lipids were fixed for 60’ in 1% osmium tetraoxide (EMS Electron Microscopy Sciences cat#19440) in cacodylate buffer at room temperature. The samples were then rinsed many times in cacodylate and in Braun water, dehydrated in increasing concentrations of ethanol, of which the 75% step contained 1% uranyl acetate. After several passages in pure 100% ethanol and two passages in pure propylene oxide (Sigma), the samples were infiltrated in increasing concentrations of epoxy resin (Agar), flat-embedded in pure epoxy resin and cured at 60°C for 48 h.

Flat-embedded human graft vertical samples were then cut transversely on an ultramicrotome (Leica UCT) starting from the graft surface and descending in steps into the cortical tissue of the host mouse, forming a “vertical probe” (Bourgeois et al., 1994). During the descent in this vertical probe, the numbers and thicknesses of all sections were registered/counted to know the depth position of the sections selected every 50 μm for observation. At these different levels of this descent, semi-thin (1 μm thick) and ultra-thin (70 nm thick) sections were obtained. The semi-thin sections were stained with toluidine blue for histological examination of the graft. The ultra-thin sections, collected on single-slot grids, were counter-stained with uranyl acetate (4% in water for 40 min, Merck) and then with lead citrate. They were examined in a transmission electron microscope (Philips Tecnai G2 at 120 kV, and the images were acquired with a Gatan Ultrascan 4000), to identify human GFP + axons and synaptic profiles.

## Data availability statement

The datasets presented in this study can be found in online repositories. The names of the repository/repositories and accession number(s) can be found below: NCBI GEO, GSE246427 https://www.ncbi.nlm.nih.gov/geo/query/acc.cgi?acc=GSE246427.

## Ethics statement

The studies involving humans were approved by the Institut Pasteur CRT Comité de Recherche Translationnelle. The studies were conducted in accordance with the local legislation and institutional requirements. The human samples used in this study were gifted from another research group. Written informed consent for participation was not required from the participants or the participants’ legal guardians/next of kin in accordance with the national legislation and institutional requirements. The animal study was approved by Local Ethics Committee CETEA 89.

## Author contributions

CR: Writing—original draft, Formal analysis, Investigation, Methodology, Validation. DG: Investigation, Writing—review and editing. GS: Investigation, Writing—review and editing. SB: Writing—review and editing, Methodology. J-PB: Writing—original draft. BJ: Writing—original draft, Software. BS: Data curation, Writing—review and editing. LS: Investigation, Writing—review and editing. SP: Formal analysis, Investigation, Methodology, Supervision, Writing—original draft. UM: Supervision, Writing—original draft, Conceptualization, Funding acquisition, Project administration.

## Footnotes

1
http://n2t.net/addgene:40755
