# Supplementary material for: Functional maturation of human iPSC-derived pyramidal neurons in vivo is dependent on proximity with the host tissue
Source: Front Cell Neurosci. 2023 Nov 23;17:1259712. doi: 10.3389/fncel.2023.1259712 (PMC10708947; doi:10.3389/fncel.2023.1259712)
Supplement: Supplementary file 1 [file Data_Sheet_1.PDF]

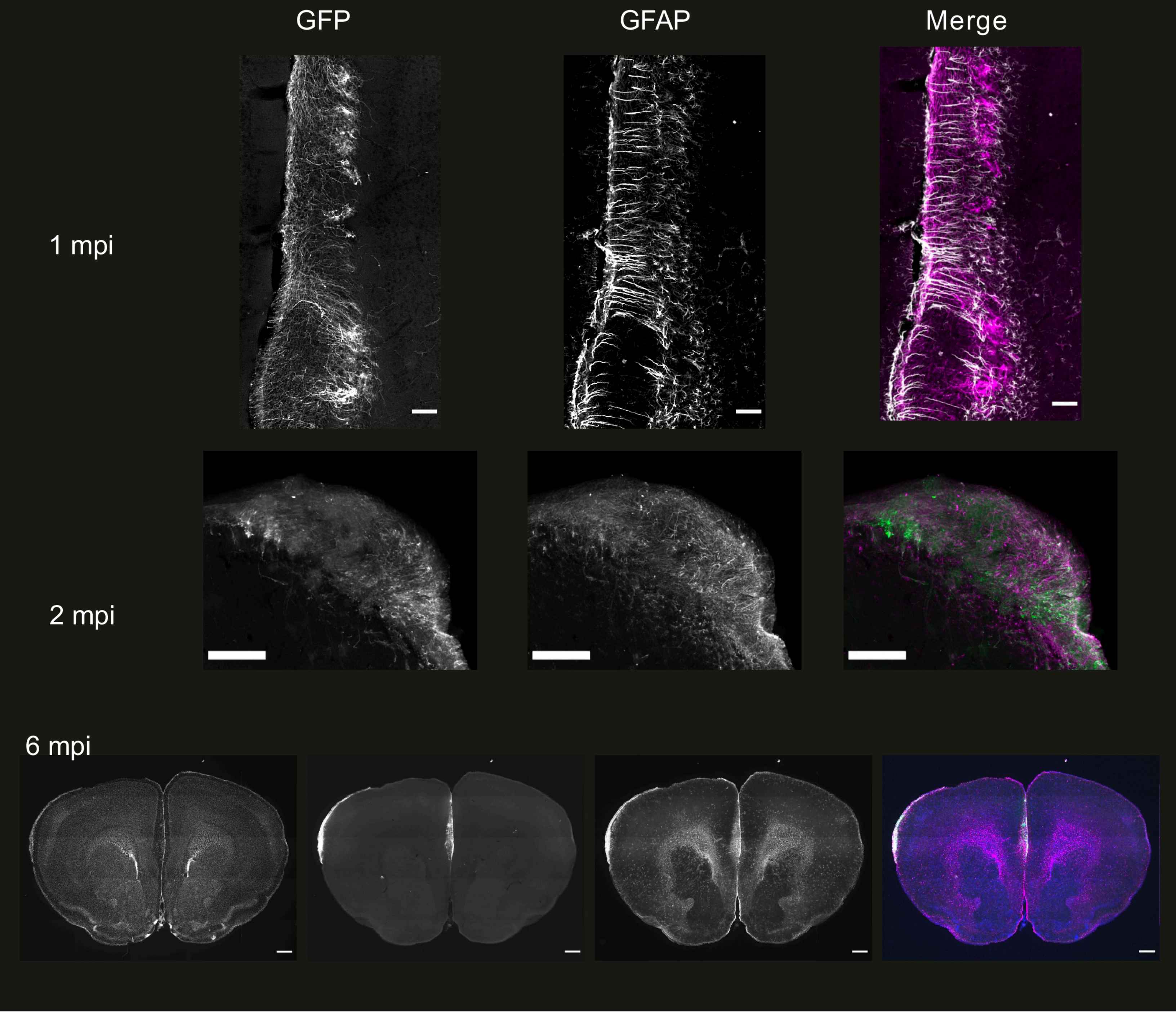

Supplementary Figure 1
Representative images of mouse brain slices 1 (A), 2 (B) and 6 (C) mpi, labeled by immunofluorescence with antibodies directed against GFP, GFAP and counterstained with DAPI.
Scale bar= 1mpi, 50µm; 2mpi, 200µm; 6mpi, 500µm.
