# Supplementary material for: Functional maturation of human iPSC-derived pyramidal neurons in vivo is dependent on proximity with the host tissue
Source: Front Cell Neurosci. 2023 Nov 23;17:1259712. doi: 10.3389/fncel.2023.1259712 (PMC10708947; doi:10.3389/fncel.2023.1259712)
Supplement: Supplementary file 2 [file Data_Sheet_2.PDF]

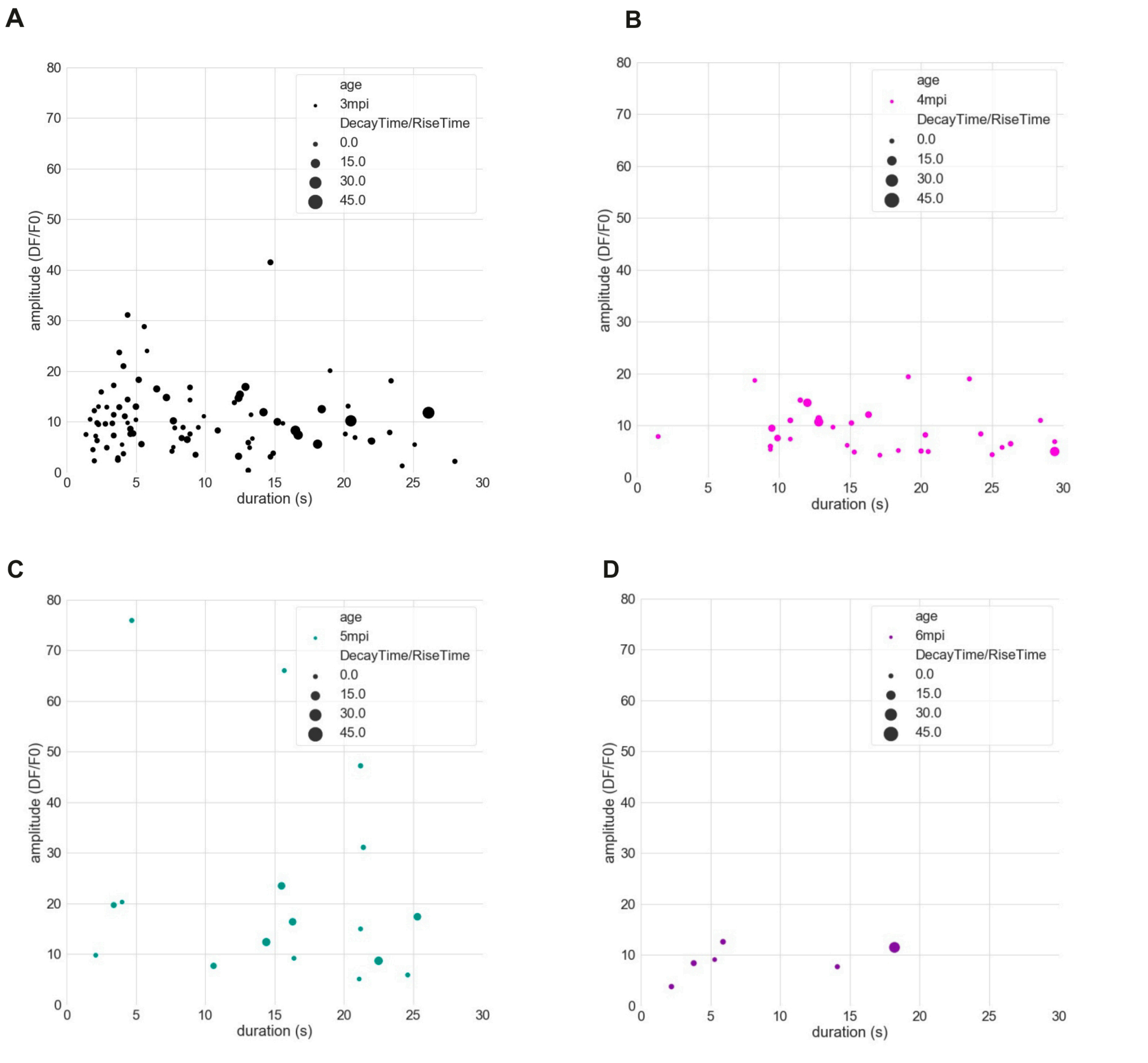

Supplementary Figure 3
Raster plot representation of the evolution of calcium transient parameters for the neurite ROIs at each time point. Each point represents one calcium transient, the position on the x-axis represents the duration of the transient, the position on the y-axis represents the amplitude of the transient, the size of the spots represents the Decay time/Rise time ratio.
