# Supplementary material for: Functional maturation of human iPSC-derived pyramidal neurons in vivo is dependent on proximity with the host tissue
Source: Front Cell Neurosci. 2023 Nov 23;17:1259712. doi: 10.3389/fncel.2023.1259712 (PMC10708947; doi:10.3389/fncel.2023.1259712)
Supplement: Supplementary file 3 [file Data_Sheet_3.PDF]

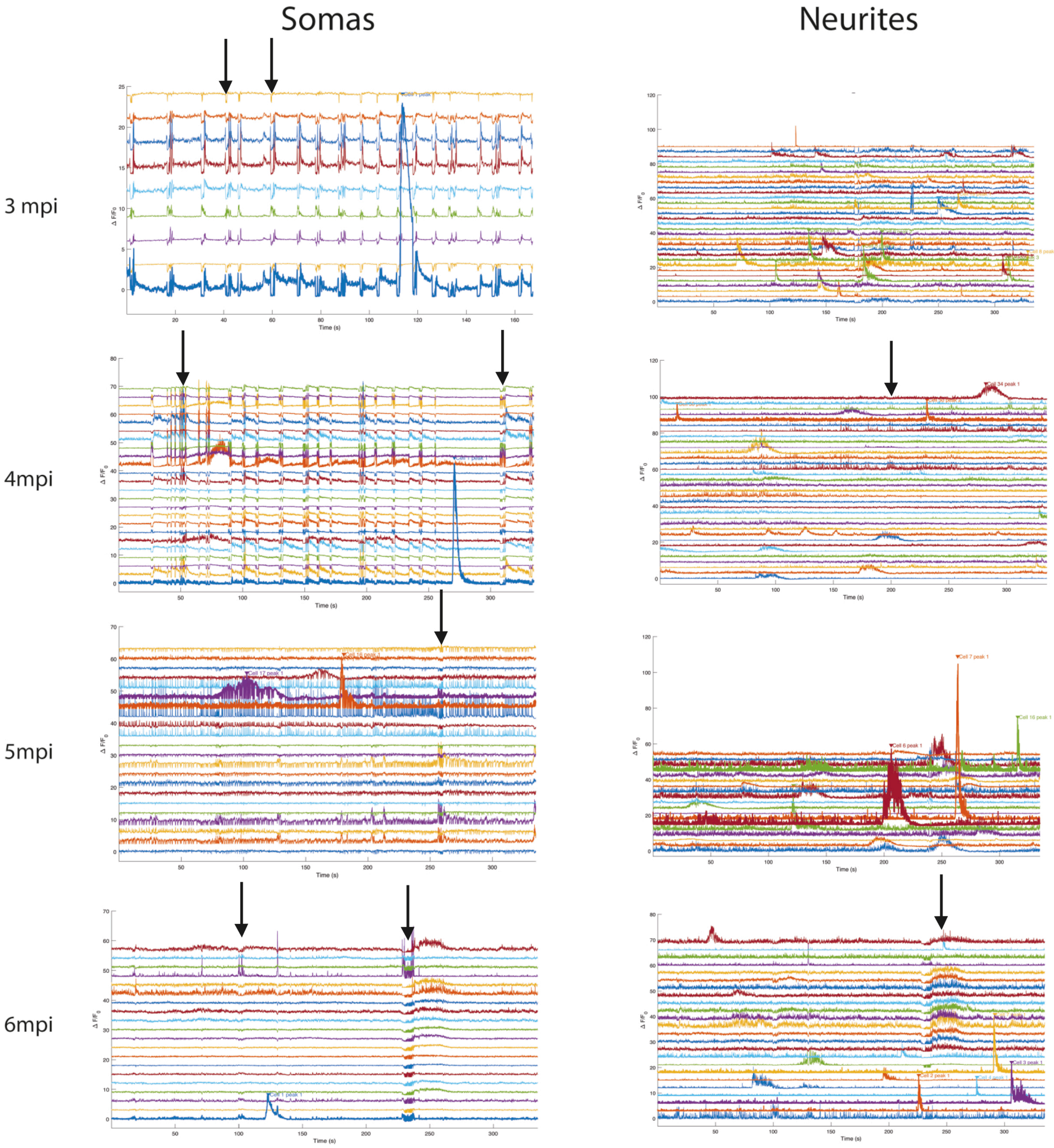

Supplementary Figure 2

Representative traces of calcium signals in active ROIs, obtained with chronic 2P microscopy at 3, 4, 5, and 6mpi, in fields of view with active soma (left column) or neurites (right column). Each graph represents the variations in fluorescence, expressed as DF/F, recorded in a given field of view, while each line corresponds to an ROI. Black arrows point to typical movement artefacts, ignored by the program as they are repeated in each ROI of the same field of view.
