# Supplementary material for: Functional maturation of human iPSC-derived pyramidal neurons in vivo is dependent on proximity with the host tissue
Source: Front Cell Neurosci. 2023 Nov 23;17:1259712. doi: 10.3389/fncel.2023.1259712 (PMC10708947; doi:10.3389/fncel.2023.1259712)
Supplement: Supplementary file 4 [file Table_1.docx]

**Supplementary Table 1**

| **Primary Antibodies** | | | | |
| --- | --- | --- | --- | --- |
| **Target** | **Host** | **Dilution** | **Source** | **Reference** |
| Bassoon | Mouse | 1/200 | abcam | Abcam Cat# ab82958, RRID:AB_1860018 |
| CD31 | Rabbit | 1/800 | Abcam | Abcam Cat# ab124432, RRID:AB_2802125 |
| CtiP2 | Rat | 1/200 | abcam | Abcam Cat# ab18465, RRID:AB_2064130 |
| CUX1 | Mouse | 1/500 | abcam | Abcam Cat# ab54583, RRID:AB_941209 |
| GAD65/67 | Rabbit | 1/1000 | Sigma-Aldrich | Sigma-Aldrich Cat# G5163, RRID:AB_477019 |
| GFAP | Rabbit | 1/400 | Abcam | Abcam Cat# ab7260, RRID:AB_305808 |
| GFP | Rabbit | 1/750 | Invitrogen | Thermo Fisher Scientific Cat# A-6455,  RRID:AB_221570 |
| GFP | Chicken | 1/1000 | Abcam | Abcam Cat# ab13970, RRID:AB_300798 |
| Homer | Rabbit | 1/500 | Synaptic systems | Synaptic Systems Cat# 160 003, RRID:AB_887730 |
| Iba1 | Rabbit | 1/1000 | Wako | FUJIFILM Wako Shibayagi Cat# 019-19741,  RRID:AB_839504 |
| MBP | Chicken | 1/100 | Millipore | Millipore Cat# AB9348, RRID:AB_11213157 |
| NeuN | Mouse | 1/200 | Millipore | Millipore Cat# MAB377, RRID:AB_2298772 |
| Olig2 | Rabbit | 1/1000 | Abcam | Abcam Cat# ab109186, RRID:AB_10861310 |
| Sox2 | Rat | 1/200 | eBioscience | Thermo Fisher Scientific Cat# 14-9811-82,  RRID:AB_11219471 |
| STEM101 | Mouse | 1/100 | Takara Bio | Takara Bio Cat# Y40400, RRID:AB_2895096 |
