# Supplementary material for: Functional maturation of human iPSC-derived pyramidal neurons in vivo is dependent on proximity with the host tissue
Source: Front Cell Neurosci. 2023 Nov 23;17:1259712. doi: 10.3389/fncel.2023.1259712 (PMC10708947; doi:10.3389/fncel.2023.1259712)
Supplement: Supplementary file 5 [file Table_2.docx]

| **Secondary Antibodies** | | | | | |
| --- | --- | --- | --- | --- | --- |
| **Target** | **Host** | **Fluorochrome** | **Dilution** | **Source** | **Reference** |
| Rabbit | Goat | Alexa Fluor 488 | 1/400 | Invitrogen | Thermo Fisher Scientific Cat# A-  11008, RRID:AB_143165 |
| Chicken | Goat | Alexa Fluor 488 | 1/1000 | Invitrogen | Molecular Probes Cat# A-11039, RRID:AB_142924 |
| Mouse | Goat | Alexa Fluor 488 | 1/200 | Life Technologies | Molecular Probes Cat# A-11029, RRID:AB_2534088 |
| Rabbit | Goat | Alexa Fluor 594 | 1/400 | Invitrogen | Thermo Fisher Scientific Cat# A- 11012, RRID:AB_2534079 |
| Mouse | Goat | Alexa Fluor 594 | 1/400 | Invitrogen | Molecular Probes Cat# A-11005,  RRID:AB_141372 |
| Rat | Donkey | Alexa Fluor 594 | 1/400 | Jackson ImmunoResearch | Jackson ImmunoResearch Labs  Cat# 712-585-150, RRID:AB_2340688 |
| Chicken | Donkey | Cy3 | 1/200 | Sigma-Aldrich | Millipore Cat# AP194C,  RRID:AB_92679 |
| Rabbit | Donkey | Alexa Fluor 647 | 1/200 | Jackson ImmunoResearch | Jackson ImmunoResearch Labs Cat# 711-605-152,  RRID:AB_2492288 |
| Mouse | Donkey | Alexa Fluor 647 | 1/200 | Jackson ImmunoResearch | Jackson ImmunoResearch Labs Cat# 715-605-150,  RRID:AB_2340862 |
| Rabbit | Donkey | Dy Light 405 | 1/200 | Jackson ImmunoResearch | Jackson ImmunoResearch Labs Cat# 711-475-152,  RRID:AB_2340616 |

**Supplementary Table 2:**
